# Modeling Organic Agriculture Expansion in the EU: Assessing Productivity and Environmental Trade‐Offs

**DOI:** 10.1111/gcb.70973

**Published:** 2026-07-13

**Authors:** Anna Muntwyler, Emanuele Lugato, Panos Panagos, Laura Scherer, Adrian Muller, Stephan Pfister

**Affiliations:** ^1^ European Commission, Joint Research Centre (JRC) Ispra Italy; ^2^ Institute of Environmental Engineering ETH Zurich Zurich Switzerland; ^3^ Agroscope, Agroecology and Environment, Agroscope (Reckenholz) Zurich Switzerland; ^4^ Institute of Environmental Sciences (CML) Leiden University Leiden the Netherlands; ^5^ Research Institute of Organic Agriculture FiBL Frick Switzerland

**Keywords:** DayCent, fertilization, freshwater eutrophication, nutrients, soil degradation, yield gap

## Abstract

Intensive agriculture has led to significant soil degradation and nutrient surpluses in the EU, prompting the need for more sustainable practices. Organic agriculture is often considered a strategy to produce food more sustainably, albeit with challenges such as yield reduction. Here, we estimate the impacts of expanding organic agriculture to 25% of agricultural land in the EU and UK—a key target of the European Commission's Farm to Fork Strategy—using a spatially explicit biogeochemical model, focusing on changes in crop yields in combination with C, N, and P fluxes and stocks. Achieving the 25% target could improve 0.8%–1.4% of degraded soil areas (out of ~49.0 million ha that exceed at least one degradation criterion based on N surplus, excess soil P, or soil erosion thresholds), quantifiably reduce dependency on mineral fertilizers (P by 15.8%–16.0% and N by 15.2%–15.7%), and either lessen or maintain current eutrophication impacts on freshwater fish. However, these benefits come with a trade‐off of about 6.5% reduction in average yields of grain and tuber, partly due to increased fodder production replacing grain and tuber crops in the rotation. Applying the 25% target area EU‐wide or per member state minimally affects the overall results. An additional cover crop scenario demonstrated the benefits of increased N fixation, improved yields, and mitigating SOC decline, but also resulted in higher impacts on freshwater biodiversity due to increased N losses. Thus, it highlights the importance of considering interconnected N, P, and C cycles alongside crop yields and potential feedbacks. This approach offers valuable insights into the synergies and trade‐offs between agricultural practices and environmental consequences at high spatial resolution.

## Graphical abstract

Expanding organic farming across the EU and UK could cut fertilizer use and improve some degraded soils, but comes with lower crop yields—driven largely by crop rotation shifts—and a modest decline in soil carbon. Increased use of nitrogen‐fixing cover crops could help maintain soil carbon and boost yields, but at the cost of greater nitrogen losses to waterways.

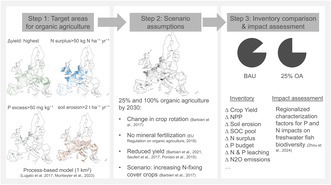

## 1 Introduction

Agriculture significantly affects soil health, which in turn is essential for sustainable food production (Barbieri et al. 2021; Pravalie et al. 2024). Intensive agricultural practices have led to soil degradation, including high nutrient surpluses, exacerbated soil erosion, depleted soil organic carbon (SOC) stocks, and biodiversity loss, resulting in an estimated 62% of EU soils being classified as unhealthy (Barbieri et al. 2021; European Parliament 2024). At the same time, nutrient surpluses (i.e., higher inputs of nitrogen (N) and phosphorus (P) than outputs through harvest) are responsible for about 80% of the global ocean and freshwater eutrophication and are likely to increase in the near future (Poore and Nemecek 2018; Zhou et al. 2024). These N and P flows from fertilizers and manure to agricultural soils are exceeding global and regional planetary boundaries (Richardson et al. 2023). Consequently, developing environmentally sustainable and resilient production systems has become an urgent priority (Ponisio et al. 2015a).

Organic agriculture (OA) is often considered a strategy to produce food more sustainably than conventional agriculture (Barbieri et al. 2021; Seufert et al. 2017; Seufert and Ramankutty 2017a). Defined by various regulations and certification guidelines, it prohibits, for example, the use of synthetic fertilizers and pesticides (Seufert et al. 2017; IFOAM 2014). OA offers potential benefits such as higher biodiversity, reduced soil erosion, improved resource efficiency, and improved soil and water quality (Seufert et al. 2017; Mäder et al. 2002; Krause et al. 2024; Seufert and Ramankutty 2017b). However, the transition from conventional to OA can also come with potential costs, such as decreased yield (Seufert 2019) that can lead to increased greenhouse gas emissions per unit crop produced (Kirchmann 2019; Meier et al. 2015; Seufert et al. 2012), land use change from the increased land requirement to produce the same amount of crops (Meier et al. 2015), or the risk of reduced net SOC stocks (Gaudaré et al. 2023). Additionally, switching from synthetic to organic nutrient inputs does not necessarily result in reduced N or P losses (Kirchmann and Bergström 2001). While these trade‐offs are important to consider, a recent systematic review of Life Cycle Assessment (LCA) data suggests that, on average, OA performs better on a per‐area basis and similarly to conventional agriculture on a per‐unit product level, though results vary widely (Hashemi et al. 2024).

Despite the ongoing scientific debate and analysis of potential trade‐offs, EU policies such as the European Green Deal's Farm to Fork strategy aim at 25% of the EU's agricultural land to be organic by 2030 (European Commission 2020a). The effects of a substantial upscaling have been assessed in recent studies using spatially explicit biophysical, biogeochemical, or empirical models on a global or European scale (Barbieri et al. 2021; Gaudaré et al. 2023; Bremmer et al. 2021; Billen et al. 2024). These studies suggest possible reductions in global C inputs and SOC stocks and severe N limitations that could drastically reduce crop yields and, thus, increase the land required compared to conventional agriculture. However, these models did not consistently account for the interconnected effects of C, N, and P cycles and climate change interactions under changed management. Additional knowledge gaps exist in the quantification of biological N fixation by legumes in the organic crop rotations, N_2_O emissions per area, N and P losses, and soil erosion (Seufert and Ramankutty 2017b).

Here, we employ the spatially explicit process‐based biogeochemical model DayCent to investigate these topics. The model has demonstrated its potential to adequately simulate crop yields, soil C, N, and P dynamics, as well as N_2_O and CO_2_ emissions, particularly under temperate conditions after data‐driven calibration (Lugato, Smith, et al. 2018; Lugato et al. 2017; Muntwyler et al. 2024; Quemada et al. 2020; Pacifico et al. 2024). It is particularly useful for simulating changing climates and management scenarios in a spatially explicit manner. Therefore, it provides insights for assessing mitigation options and policy goals.

While biogeochemical element flows and stocks indicate environmental pollution risk and resource management (Panagos et al. 2024; Einarsson et al. 2020; Jedelhauser and Binder 2015; Panagos, Köningner, et al. 2022), LCA complements these indicators by linking emissions and resource use to impacts through a cause‐effect chain involving fate and effect models (Rosenbaum et al. 2018). Recently developed regionalized characterization factors (CFs) address freshwater eutrophication by linking nutrient concentrations to species loss of freshwater fish, considering various pathways of N and P losses and the limiting nutrients of the water body (Zhou et al. 2024). By integrating these CFs, our assessment captures freshwater biodiversity impacts, enabling a more comprehensive evaluation of OA's environmental trade‐offs.

The objectives of this study are to estimate the potential impacts of expanding OA to cover 25% of the agricultural land area in the EU and UK (a former EU member) using the spatially explicit, process‐based DayCent model. By focusing on changes in crop yields, in combination with changes in the fluxes and stocks of C, N, and P, we identified areas where transitioning to OA would simultaneously minimize soil degradation (and the associated environmental pollution) and crop yield reductions. The study compared the OA scenario to a business‐as‐usual (BAU) scenario and integrated the biogeochemical fluxes into an impact assessment using spatially explicit CFs for freshwater eutrophication.

## 2 Materials and Methods

### 2.1 Model Framework

#### 2.1.1 Process‐Based Biogeochemical Model

The process‐based ecosystem model DayCent simulates the daily dynamics of C, N, and P across the soil–plant‐atmosphere interfaces through integrating submodels for plant growth, organic matter decomposition, soil water and temperature, mineralization of soil C, N, and P, and N gas fluxes resulting from nitrification and denitrification (Hartman et al. 2018; Parton et al. 1988; Del Grosso et al. 2009). They are driven by site‐specific soil characteristics, daily meteorology, and agricultural management practices. Once calibrated and validated, the model can assess current and future changes in plant growth and soil biogeochemistry in response to changes in climate and management practices. Further model details are provided in the Supporting Information (Section 1).

#### 2.1.2 Model Parameterization

We used the DayCent version DD13centEVI over a 1‐km^2^ grid as calibrated by Muntwyler et al. (2024). The DayCent model framework has been calibrated and validated for key processes in previous studies, including the carbon cycle (Lugato, Smith, et al. 2018; Lugato et al. 2014, 2016, 2020; Lugato, Leip, and Jones 2018), erosion (Lugato, Smith, et al. 2018; Lugato et al. 2016), yield prediction (Pacifico et al. 2024), and the N (Lugato, Smith, et al. 2018; Lugato et al. 2017; Quemada et al. 2020; Pacifico et al. 2024) and P (Muntwyler et al. 2024, 2023) cycles using data from European long‐term field experiments and large scale datasets. The framework was subsequently modified to represent an increased share of OA (see Section 2.2). As in the previous studies, the starting year of the simulation was set in 1980, allowing 30 years for all fast and slow nutrient pools to reach equilibrium. The initial SOC pool partitioning was derived from a long‐term spin‐up (Lugato et al. 2014). An overview of the data inputs, their spatial resolution, and the model outputs to run the model are summarized in Figure S1. All inputs were harmonized to the same spatial resolution.

The meteorological data (daily minimum and maximum temperature and precipitation) are taken from the E‐OBS gridded dataset (www.ecad.eu/). The soil characteristics, including initial nutrient pools, bulk density, soil pH, soil texture, and derived hydraulic properties, were based on the chemical properties maps derived from approximately 20,000 measured soil samples of the Land Use and Cover Area frame Survey (LUCAS) (Ballabio et al. 2019; Orgiazzi et al. 2018). The mineral nutrient inputs were derived from the decadal average national mineral fertilizer consumption data from EUROSTAT, allocated to the agricultural areas based on the theoretical agronomic requirements of the crops, with preferential allocation given to arable land, while pastures received the remaining mineral fertilizer (maximum up to 10% of the total mineral P fertilizer). Organic C, N, and P inputs were derived from FAO livestock density maps using species‐specific excretion coefficients (see Section 1e in Supporting Information). The average wet and dry N depositions were sourced from the EMEP model (rv 4.5).

The historic and current land use was based on the Corine Landf Cover database (1990, 2000, 2006, 2012), encompassing the areas defined as arable land, rice, pasture, and heterogenous areas. The study area, thus, included an area of about 158 million ha. We adopted the crop parametrization from previous work (Muntwyler et al. 2024; Lugato, Leip, and Jones 2018; Lugato et al. 2016), following crop production statistics, management calendars, and the calibrated crop parameters maximum potential growth rate (PRDX), the harvest index (HIMAX), and CN and CP ratios, differentiating 18 arable/fodder crops. A representative four‐year rotation was constructed for the identified arable land using NUTS2‐level average crop area statistics from Eurostat (2018). The planting and harvesting dates for each crop were derived from regional crop calendar map that is available at the SAGE Center (Sacks et al. 2010). The agricultural management practices (i.e., crop rotation, grazing, tillage type, cover crop presence, and irrigated areas) were also retained from the previously published studies. The practices were derived from Farm Structure Surveys of 2010 and 2016 (https://ec.europa.eu/eurostat/web/microdata/farm‐structure‐survey) and FAO‐AQUASTAT (Siebert et al. 2005), respectively.

#### 2.1.3 Nutrient Stocks and Flows at High Resolution

All nutrient stocks and flows are model outputs. The carbon (C) cycle is assessed by examining changes in SOC stocks resulting from the C input into the soil through different agricultural practices and their outputs. The soil C budget includes the fluxes:

(1) $$\begin{array}{c} C\;\text{budget}=C_{\text{Manure}}+C_{\text{Residue}\&\text{Roots Not Removed}}-C_{\text{Soil Respiration}}\\ \;-C_{\mathrm{Org}\;\text{Leaching}}-\mathrm{Net}\;C_{\text{Erosion}} \end{array}$$

The C input into the soil was also assessed by considering only the input terms. See Table S1 for full parameter descriptions.

P budgets are also useful to monitor whether the applied P is utilized efficiently or accumulates in the soil, thereby posing environmental pollution risks. The P budget is calculated as follows:

(2) $$\begin{array}{c} P\;\text{budget}=P_{\text{Mineral Fertilizer}}+P_{\text{Manure}}+P_{\text{Chemical Weathering}}\\ \;-P_{\text{Crop Harvest}}-P_{\text{Residue Removal}}-\mathrm{Net}\;P_{\text{Erosion}}\\ \;-P_{\mathrm{Org}\;\text{Leaching}} \end{array}$$

Additionally, the simulated soil plant‐available P is examined to identify areas where shifting towards OA could be beneficial, as detailed in the following section. Unlike P, the N surplus does not accumulate in the soil, comprising forms of reactive N that are prone to leaching and gaseous losses. Therefore, the gross N budget calculated as all N inputs minus N outputs via crop harvest is used to compare the management practices and select the areas beneficial for shifting:

(3) $$\begin{array}{c} \text{Gross}\;N\;\text{budget}=N_{\text{Mineral Fertilizer}}+N_{\text{Manure}}+N_{\text{Fixation}}\\ \;+N_{\text{Deposition}}-N_{\text{Crop Harvest}}-N_{\text{Residue Removal}} \end{array}$$

Other N losses, such as N leaching (organic & mineral), N erosion, N_2_O, and NO_x_, are not included in the budget equation but are reported and included in the comparison. These fluxes indicate how surplus N gets distributed in the environment, providing insight into environmental impacts. Leaching and erosion of N and P leaching are linked to the environmental impact assessment (see Section 2.2). All individual N fluxes are provided in the dataset, enabling users to derive a net N budget, if desired.

The net soil erosion and its nutrient‐specific counterparts, C_Erosion_, P_Erosion_, and N_Erosion_, were calculated by coupling DayCent with the RUSLE semi‐empirical model, which accounts for water erosion through runoff, sheet, and rill erosion (Lugato, Smith, et al. 2018; Lugato et al. 2016; Panagos et al. 2015). In the simulation, erosion was implemented as a single event in autumn based on RUSLE's average annual erosion rate. A parsimonious soil deposition approach was applied by multiplying the gross soil erosion with the spatially explicit sediment delivery ratio from Borrelli et al. (2018). to get net soil erosion and thus sediments, N, and P, entering the riverine system as previously done in Muntwyler et al. (2024). However, to identify the areas degraded by soil erosion by water, gross erosion was obtained from Panagos et al. (2020).

Other potential inputs (such as externally produced compost, sewage sludge, planting material, atmospheric P deposition) or outputs (such as losses through tillage‐ and wind‐induced erosion) of C, N, or P were not considered significant compared to the included variables in Equations ((1), (2), (3)) or not included due to the scarcity of data at continental scale (Huygens et al. 2022; Viaene et al. 2016; Lun et al. 2018). The nutrient and C pools of the topsoil (0–30 cm) and the flows reported are averaged over a four‐year crop rotation.

### 2.2 Business as Usual and Organic Agriculture Expansion Scenarios

The model was run from 1980 until 2022 with the described model parameterization. The period from 2019 to 2022 represents the current state of agricultural management and biogeochemical cycles. From 2023 onwards, the model was extended to project a business‐as‐usual (BAU) scenario alongside scenarios with changed agricultural management until 2034 (with OA vs. BAU comparisons referring to the 2031–2034 rotation period). The bias‐corrected climatic projections were based on the general circulation model CNRM‐CM541 and the Representative Concentration Pathway 4.5 (RCP4.5) climate scenario (http://www.ecad.eu), with a CO_2_ fertilization effect incorporated in the model. While management practices and crop rotations were modified to simulate OA expansion within the agricultural areas, no land use changes were implemented. The study focused on what‐if scenarios within existing agricultural land, isolating the effects of converting from conventional to organic management.

**FIGURE 1:**
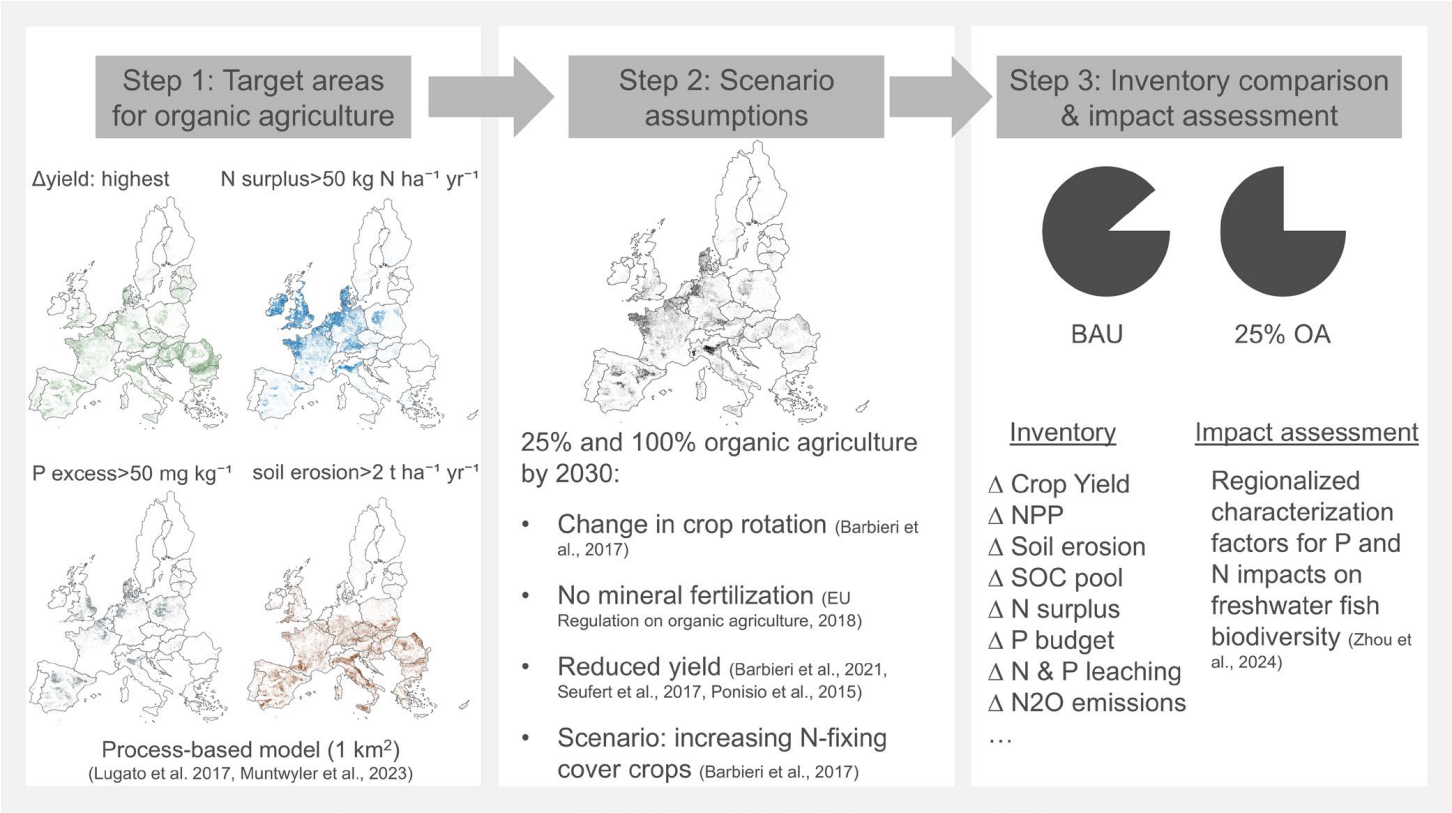
Framework diagram detailing the methodology of this study, including the criteria for selecting the target area for transitioning to organic agriculture, the transition assumptions, and the comparison of the business‐as‐usual scenario with an increased organic agriculture scenario (100% or 25% OA) using life cycle assessment. The target area optimal for changing to OA was determined by the projected yield, as well as soil health indicators (N surplus > 50 kg ha^−1^ year^−1^, P excess > 50 mg kg^−1^, soil erosion > 2 t ha^−1^ year^−1^) at a spatial resolution of 1 km^2^. Map lines delineate study areas and do not necessarily depict accepted national boundaries.

The BAU management was contrasted with a scenario that increased the share of OA by implementing a series of changes in agricultural management practices. Figure 1 summarizes the methodological framework of this study. Firstly, to achieve the target of 25% OA coverage across the EU, we targeted specific areas for conversion, as detailed below. This approach was applied both (a) EU‐wide and (b) within each member state and the UK. The current share of organic crop area was sourced from (EUROSTAT 2024), including “total fully converted and under conversion to organic farming” and “excluding kitchen gardens” for the year 2022 for all EU countries and the UK for which the data from 2019 was used. Areas “currently in transition to organic agriculture” were included in the current OA area, as this process necessitates immediate changes in management practices, such as the prohibition of synthetic pesticides. To determine the required increase in OA, we subtracted the current share of OA from the 25% target and multiplied the resulting percentage by the total agricultural area defined in this study (Table S2).

The criteria for transitioning conventional agriculture to OA were determined based on areas experiencing soil degradation, as well as areas with the smallest reduction in grain and tuber yields, to limit impacts on food production. Grain and tuber yields were simulated by the model and converted from C (the output metric of the DayCent for crop production) to dry matter. The areas experiencing soil degradation were identified by soil health indicators, including the risk of N losses (BAU N surplus > 50 kg ha^−1^ year^−1^), excess soil P (available soil *p* > 50 mg kg^−1^), and soil erosion (stemming from water erosion > 2 t ha^−1^ year^−1^) (Panagos et al. 2024; European Parliament 2024). In areas where the soil has sufficient legacy P available for plants, the use of the critical raw material phosphate rock is an inefficient and unnecessary resource use to sustain crop production (European Commission 2020b). Similarly, the application of additional mineral N fertilizers, which require fossil fuel inputs for their production with associated climate change impacts (Oberle et al. 2019) is environmentally inefficient in areas already experiencing N surplus. Areas with high soil erosion by water, a leading cause of soil degradation in the EU (Panagos et al. 2021), are targeted to decrease nutrient losses through erosion and maintain overall soil quality. This targeted transition approach focuses on areas where sufficient organic nutrient resources (e.g., manure, legacy P) already exist, thereby avoiding the need for external inputs or new infrastructure.

The transitioning area varied due to the unique conditions concerning the extent of soil degradation and the degree of yield reduction calculated at the pixel level. The area was selected in sequence: first, areas exceeding one or more soil health thresholds, and secondly, areas exhibiting the smallest potential yield reductions when converted to OA. This approach identified areas where a conversion has a high effect on soil health while maximizing yield, thus favoring the transition in croplands. It was applied EU‐wide, allowing for a simultaneous application of the criteria, or per member‐state (see details in Section 2.3 in Supporting Information). The mineral N and P fertilizer input was set to zero, as organic regulations prohibit the use of chemical fertilizers, while the organic manure input was kept constant, a conservative livestock scenario.

Significant differences in crop rotations between conventional and organic farming have been documented, varying by global regions (Barbieri et al. 2017, 2019). In Europe, primary and secondary cereals are far less abundant in organic rotations, while grain pulses and temporary fodder are increasingly grown. Thus, we adapted the crop rotations following the time‐shares found by Barbieri et al. (2019) for Europe per NUTS2 (Table S2), maintaining a four‐year duration. The decrease in cereal production was offset by the simulation of pulses and temporary fodder on the released agricultural land, with these crops occupying 40% and 60% of the area, respectively. For temporary fodder, a one year N‐fixing grass‐clover mix from Lugato et al. (2015) was selected since N‐fixing crops are more abundant in organic farming. Barbieri et al. (2017) also concluded that N‐fixing catch and cover crops are much more abundant in organic crop rotations than in conventional ones. Thus, we included an additional scenario where N‐fixing, soil erosion‐reducing cover crops (vetch) are introduced as “green manure” between the harvest and planting of two consecutive main crops when at least two months intervened (Alewell et al. 2020; Giordano et al. 2021; Rivière et al. 2022).

DayCent simulates the effects of nutrient management changes on crop yields (grain and tuber) and net primary productivity (NPP). However, the ban on synthetic pesticides in OA leads to a yield reduction due to increased pests, diseases, and weeds (Seufert and Ramankutty 2017b). While the model does not directly incorporate this yield gap, it was simulated indirectly by adjusting the maximum potential crop productivity parameter. The presence of a yield gap is uncontested in literature, but its magnitude is debated (Barbieri et al. 2021; Seufert and Ramankutty 2017b; Seufert et al. 2012; De Ponti et al. 2012; Klinnert et al. 2024; Ponisio et al. 2015b; Wilbois and Schmidt 2019). Reported yield gaps vary depending on crop type, crop species, crop diversity in rotations, nutrient input, geographical region, and other variables. Therefore, we implemented two scenarios based on literature values for developed countries, focusing on yield gaps that are not related to nutrient limitations, but to factors such as pest and disease management and crop diversity. These high and low yield gap scenarios diminish crop yield due to changed plant protection measures by 20% and 10%, respectively. A detailed justification of these yield gap values based on literature evidence is provided in Section 2.1 in Supporting Information. For temporary grass‐clover fodder crops and grassland, the plant protection related yield gap was assumed to be zero since these typically do not receive plant protection agents (Wilbois and Schmidt 2019).

The modeling also allows for a factorial approach for the scenarios, systematically implementing one change in agricultural management practice at a time (no mineral fertilization, changed crop rotation, yield gap from changed plant protection measures, additional cover crops). This method allowed for a disaggregation of the effects of each factor, enabling an understanding of their individual and combined impacts on the results. For instance, we could analyze scenarios both with and without the yield gap from the changed plant protection measures (Tables S3–S5).

### 2.3 Assessing Eutrophication Impacts

To compare the environmental impact of OA with the BAU scenarios, we focused on their impacts on freshwater fish biodiversity, allowing for a specific assessment of the environmental consequence of transitioning to OA directly related to their nutrient managements. For this, the nutrient losses derived from the model framework are connected to their impact on freshwater fish, applying CFs for freshwater eutrophication from the LCA framework. Losses of N and P from soil erosion and leaching, expressed as kg nutrient ha^−1^, represent the inventory data. We assessed both the high and low yield gap scenarios. To identify the crop with the highest impact, the C content of the crops was converted into dry weight using a C content factor (Eurostat 2017). The inventory data was combined with recently published CFs with global coverage by Zhou et al. (2024). The CFs are based on the global nutrient model IMAGE‐GNM, which is particularly suitable for Europe (Jiao et al. 2023). These updated CFs combine spatially explicit fate factors (FF) with effect factors (EF) to estimate the global potentially disappeared fraction (PDF) of species due to N and P concentration in freshwater over a certain exposure period (year), considering the effect of eutrophication on fish species. Eutrophication in a water body can be limited by P, N, or co‐limited by both nutrients (Zhou et al. 2024). Only the nutrient‐limiting CFs were applied based on Zhou et al.'s spatially explicit map of nutrient limitation.

Two different kinds of CF exist: marginal or average. The marginal approach denotes the effect of an additional activity, while the average approach attributes the damage evenly to all emissions. The average CFs were used to calculate and present the main results (Figure 4), whereas both marginal and average CFs were employed for comparison and transparency (Figure S8). The CFs were available for direct emissions to freshwater, diffuse emissions, and increased erosion due to agricultural land use. As we considered the pathways for net erosion and diffuse sources to the water body within the DayCent model, we assumed all nutrient fluxes at this stage as direct emissions expressed as PDF·year kg^−1^ nutrient. Given that these CFs were available at a 0.5 × 0.5‐degree and the inventory at 1 km^2^ resolution, we aligned them for accurate spatial analysis using bilinear resampling. Additionally, where the direct CFs were not available at the grid‐cell level, we created national CFs averages over the nutrient‐limited areas. The impact of each scenario (PDF·years) was calculated by summing the products of the inventory values and the CFs for each source cell (i):

(4) $$\text{Impact}=\sum \left({\text{Inventory}}_{i}\times {\mathrm{CF}}_{i}\;\right)$$

## 3 Results

### 3.1 Areas Selected for Conversion to Organic Agriculture

**FIGURE 2:**
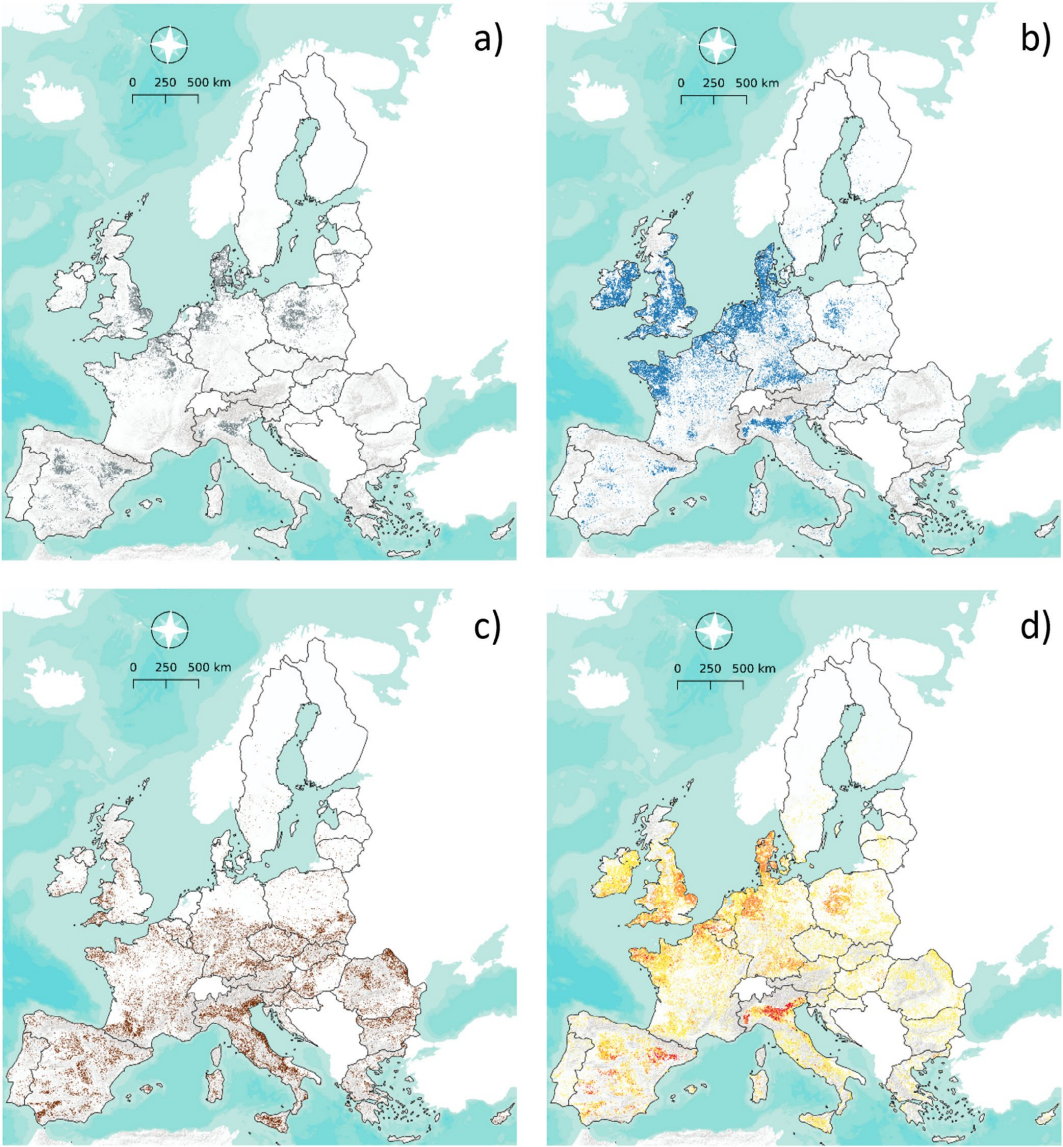
Agricultural soils affected by (a) excess soil P (available soil *p* > 50 mg kg^−1^), (b) N surplus (> 50 kg ha^−1^ year^−1^), or (c) soil erosion through water (> 2 t ha^−1^ year^−1^), and (d) whether soil degradation processes overlap in the EU and UK. Red areas indicate that 3 indicators are exceeded, and orange indicates that 2 are. Map lines delineate study areas and do not necessarily depict accepted national boundaries.

The areas designated to transition to OA were selected by the current soil degradation, determined by N surplus, excess soil P, and soil erosion thresholds, in addition to minimizing yield reduction. Across the EU and UK, approximately 49.0 million ha exceed at least one soil degradation criteria (Figure 2). About 13.9 million ha are degraded according to two indicators, and a critical 0.18 million hectares surpass all three drivers of degradation, indicating severe degradation. Countries with the largest affected areas coincide with large agricultural area, such as France (13.5 million ha), Germany (12.5 million ha), and the UK (10.5 million ha). Multiple exceedances are particularly significant in countries with intensive agriculture, such as Denmark, Belgium, Poland, and Italy, underscoring the widespread nature of soil health challenges in these regions. Achieving the 25% OA target could restore 0.8%–1.4% of the degraded soil areas by the period 2031–2034, depending on the yield reduction scenario and the application area. In all scenarios, areas exceeding one or three degradation thresholds decreased, while those exceeding two criteria increased (likely from areas exceeding three degradation thresholds).

**FIGURE 3:**
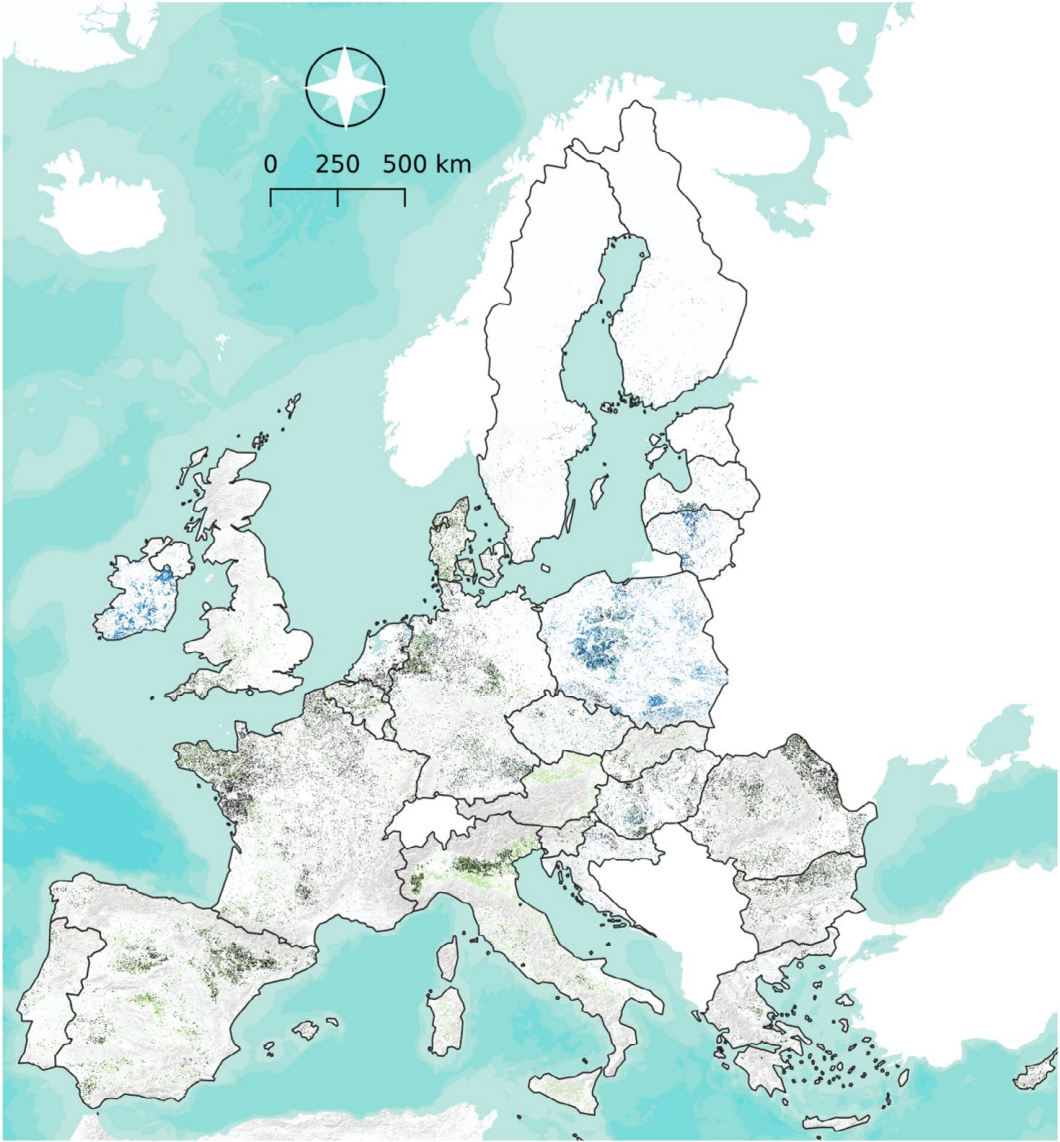
Distribution of areas selected for transition to organic agriculture under 25% target scenarios. Black: Areas selected in both EU‐wide and member state‐specific approaches (overlapping areas). Blue: Areas selected only in the member state‐specific approach. Green: Areas selected only using the EU‐wide approach. Map lines delineate study areas and do not necessarily depict accepted national boundaries.

In the EU‐wide strategy to reach 25% OA, countries with large agricultural areas, such as France (Bretagne, Pays de la Loire, Hauts‐de‐France), Italy (Lombardi, Veneto, Emilia‐Romagna), Spain (Castilla‐La Mancha, Aragón, Castilla y León) and Germany (Niedersachsen) (Figure 3), also have large areas for transition in both yield gap scenarios (see Table S3). Relative to their agricultural areas, Luxembourg, Denmark, Belgium, and Slovenia exhibited large conversion areas. The advantage of the EU‐wide approach over the member‐state approach lies in its larger pool of potential areas, allowing for more strategic choices based on soil degradation status and yield reduction. Conversely, the member‐state approach necessitated, in some countries, to select areas exceeding the soil thresholds with sub‐optimal yields to meet the national quota.

The OA transition by member state differed from the EU‐wide approach (Figure 3), influenced by the current share of OA (Table S3). Country‐level figures, including current OA shares and absolute transition areas, are provided in Table S3. The areas selected varied slightly between the two yield gap scenarios.

### 3.2 Change in Crop Yield & Fodder Production

During the twelve‐year projection, the total grain and tuber production declined across all scenarios, with differences between BAU and the increased OA scenario becoming apparent by 2031–2034. In BAU, yields decreased by 4.4% and NPP by 2.7% due to climate change affecting plant productivity and potential N limitations in certain areas, indicating current soil nutrient mining (see Figure S3). In a hypothetical scenario where all agricultural fields transition to OA, the average grain and tuber production over the 4‐year crop rotation would decrease significantly by 55.7% ± 2.3% compared to BAU (Table S4). The factorial application of the scenarios revealed the relative contributions to this yield reduction: the absence of mineral fertilization accounted for the largest contribution, followed by the effect of altered plant protection measures, and changes in the crop rotations, where high‐yielding grain crops were replaced by lower‐yielding pulses or fodder that does not contribute to grain yield. The NPP would decrease by 23.0% ± 1.3%, also primarily due to the absence of mineral fertilizers. In contrast, the changes in the crop rotation alone increased NPP. The scenario that incorporated a N‐fixing cover crop significantly increased both yield and NPP. The reason is the induced fertilization effect of the leguminous cover crop by biological N fixation, as described in Muntwyler et al. (2024).

Under an EU‐wide implementation of 25% OA, the grain and tuber yield was 1.36 ± 0.02 t C ha^−1^ year^−1^ (amounting to 214.6 Mt. C year^−1^; a 6.4% reduction relative to BAU), and the NPP 6.41 ± 0.03 t C ha^−1^ year^−1^ (2.6% less than BAU) (Table 1). The areas selected for transition exceeded at least one soil health indicator, with the lowest observed yields being 57.7% (low yield gap) or 51.3% (high yield gap) of the BAU yield. If the cover crop scenario that incorporated a N‐fixing cover crop was applied to the same 25% OA area target, the grain and tuber yield would reach 1.37 ± 0.02 t C ha^−1^ year^−1^ (equalling 216.1 Mt. C year^−1^, a 5.7% reduction). NPP would even remain 6.58 t C ha^−1^ year^−1^, equalling the same NPP as BAU Implementing 25% OA at a member‐state level resulted in reductions in NPP and total grain and tuber production similar to those observed when the entire EU and the UK agricultural area was eligible for selection (Table 1). This similarity likely stems from the application of consistent rules and the selection of identical or similar grid cells across both approaches. When cover crops were incorporated into the OA scenarios, NPP did not decrease relative to the BAU scenario, surpassing the NPP in the OA scenario without cover crops.

**TABLE 1:** Average carbon production, nutrient fluxes, and stocks under BAU and 25% organic agriculture.

Variable	Unit	BAU	EU‐wide	EU‐wide incl. cover crops	Member‐state	Member‐state incl. cover crops
NPP C	t ha^−1^ year^−1^	6.58	6.41 (−2.6%)	6.58 (−0%)	6.41 (−2.6%)	6.58 (−0%)
NPP C	Mt year^−1^	1039.1	1011.6 (−2.6%)	1039.1 (−0%)	1012.0 (−2.6%)	1039.1 (−0%)
C grain & tuber	t ha^−1^ year^−1^	1.45	1.36 (−6.4%)	1.37 (−5.7%)	1.36 (−6.5%)	1.37 (−5.5%)
C grain & tuber	Mt year^−1^	229.2	214.6 (−6.4).	216.1 (−5.7)	214.2 (−6.5%)	216.7 (−5.5%)
SOC	t ha^−1^	78.2	78.1 (−0.1%)	78.2 (−0%)	78.1 (−0.1%)	78.2 (−0%)
C input	t ha^−1^ year^−1^	3.28	3.22 (−1.9%)	3.39 (+3.3%)	3.22 (−1.9%)	3.4 (+3.6%)
Net soil erosion	t ha^−1^ year^−1^	0.42	0.41 (−1.9%)	0.4 (−4.2%)	0.42 (−1.5%)	0.41 (−3.1%)
Net C erosion	kg ha^−1^ year^−1^	8.68	8.52 (−1.8%)	8.41 (−3.1%)	8.57 (−1.3%)	8.49 (−2.1%)
C respiration	kg ha^−1^ year^−1^	3.28	3.22 (−1.8%)	3.38 (+3.1%)	3.23 (−1.7%)	3.4 (+3.6%)
C budget	kg ha^−1^ year^−1^	−15.06	−20.57 (+36.6%)	−9.54 (−36.6%)	−20.06 (+33.2%)	−14.55 (−3.4%)
Soil P total	kg ha^−1^	2246.5	2240.8 (−0.3%)	2239.8 (−0.3%)	2240.6 (−0.3%)	2239.4 (−0.3%)
Soil P available	kg ha^−1^	714.2	701.7 (−1.8%)	693.3 (−2.9%)	701.0 (−1.9%)	691.5 (−3.2%)
P budget	kg ha^−1^ year^−1^	0.28	−0.15 (−154.7%)	−0.19 (−168.3%)	−0.16 (−158.2%)	−0.22 (−178.9%)
P leaching	kg ha^−1^ year^−1^	0.005	0.0047 (−2.3%)	0.0048 (+0.7%)	0.0047 (−2.2%)	0.0048 (+1.1%)
Mineral P input	kg ha^−1^ year^−1^	6.36	5.34 (−16%)	5.35 (−15.8%)	5.35 (−15.8%)	5.36 (−15.6%)
Net P erosion	kg ha^−1^ year^−1^	0.24	0.24 (−2.3%)	0.23 (−4.7%)	0.24 (−1.7%)	0.24 (−3.4%)
Soil N total	kg ha^−1^	8443.3	8432.0 (−0.1%)	8460.6 (+0.2%)	8431.1 (−0.1%)	8460.6 (+0.2%)
Gross N budget	kg ha^−1^ year^−1^	45.86	44.62 (−2.7%)	53.06 (+15.7%)	44.48 (−3%)	52.7 (+14.9%)
Mineral N input	kg ha^−1^ year^−1^	57.72	48.92 (−15.2%)	49 (−15.1%)	48.66 (−15.7%)	48.76 (−15.5%)
N fixation	kg ha^−1^ year^−1^	15.17	20.18 (+33%)	29.11 (+91.8%)	20.31 (+33.9%)	29.18 (+92.3%)
N leaching	kg ha^−1^ year^−1^	35.35	35.20 (−0.4%)	39.00 (+10.3%)	34.94 (−1.1%)	38.81 (+9.8%)
N_2_O‐N	kg ha^−1^ year^−1^	2.32	2.25 (−3.3%)	2.48 (+6.6%)	2.24 (−3.6%)	2.45 (+5.3%)
NO_x_‐N	kg ha^−1^ year^−1^	1.93	1.88 (−2.3%)	1.99 (+3.1%)	1.87 (−3.3%)	1.99 (+3%)
N_2_	kg ha^−1^ year^−1^	3.73	4.07 (+9.2%)	4.75 (+27.4%)	4.08 (+9.5%)	4.53 (+21.6%)
Net N erosion	kg ha^−1^ year^−1^	0.95	0.93 (−1.8%)	0.92 (−3.1%)	0.93 (−1.3%)	0.93 (−2.2%)

*Note:* EU average NPP, grain and tuber C production, and nutrient fluxes and stocks under the BAU scenario and 25% organic agriculture implementation, both EU‐wide and by member state, over the whole crop rotation. The values presented here are the averages of both low/high yield gap scenarios, with the ranges shown in Table S7. Percentage values show deviations from the BAU scenario (average of 2031–2034).

### 3.3 Change in Nutrient Pools & Fluxes

In the BAU scenario, nutrient inputs remained constant relative to current conditions, but nutrient exports declined alongside decreasing crop yields. This led to marginal decreases in C inputs into the soil as no exogenous inputs of C were added, contributing to a slight decrease in both SOC and total soil N pools across the EU (Tables S4 and S5). However, the total soil P pool experienced a slight increase due to a P surplus that allowed P accumulation in the soil (Table S6). Both N_2_O emissions and N leaching were slightly increased.

Adopting OA across all agricultural areas (100% OA scenario) resulted in a decrease in SOC pool beyond the projected BAU scenario, alongside significant decreases in C inputs into the soil (Table S4). The organic crop rotation decreased soil erosion and the associated N and P losses by over 10% each (Tables S4 and S5). The drastic reduction in total N inputs (16% decrease resulting from the removal of mineral N fertilizers) led to a decrease in total soil N, N_2_O emissions, and an expansion of areas where crops are N limited (Figure S3). In contrast, N leaching and N surplus increased due to a significantly higher N fixation (by a factor of 3.6), enhanced mineralization of soil organic matter, reduced N export via grain and tuber harvests and crop residue removal, along with a potential time lag between N fixation and its availability for crop uptake. Including the cover crop scenario, N fixation rose to 7.8 times compared to BAU. This boosted crop yields, as discussed in Section 3.2, and led to a rise in N surplus (126%), resulting in increased N losses. The soil P budget shifted from a surplus balance in BAU (0.28 kg ha^−1^ year^−1^) to a P deficit of −2.0 ± 0.2 kg ha^−1^ year^−1^, driven by lower P inputs, with total average P inputs nearly halved, and lower P removal through crop harvesting (Table S6). This led to a net depletion of the average total soil P pool, a process referred to as soil P mining. However, areas predominantly fertilized with manure either maintained or increased their soil total P, as the reduced crop exportation led to P accumulation in the soil. The increased cover crop scenario further decreased the P budget due to the N supply by fixation, which increased P export through crop harvest and residue removal while also reducing P losses due to soil erosion, as described in Muntwyler et al. (2024).

Under an EU‐wide 25% OA target, not confined to individual country borders, trends mirror full OA implementation (Table 1), but show more nuanced outcomes, balancing positive and negative effects. The reduction in the SOC pool was considerably more moderate than in scenarios where organic practices are universally applied, due to smaller reductions in NPP and, consequently, C inputs. Meanwhile, the beneficial impact of organic farming reducing soil erosion was less pronounced, as fewer areas benefitted from the changed crop rotation. The P budget was the most balanced across all scenarios, nearing a net‐zero change with a corresponding decrease in P erosion. Notably, overall mineral P fertilizer use decreased by 16.0% (corresponding to 1.6 Mt. year^−1^). Similarly, mineral N fertilizer use was significantly reduced by 15.2% (corresponding to 1.4 Mt. year^−1^), but total N inputs remaining close to baseline levels due to increased N fixation in the organic scenario (Figure S5). This resulted in slight reductions in N surplus and N leaching compared to BAU, a notable improvement over the 100% OA scenario, where these values exceeded BAU levels. Gaseous emissions of N_2_O and NO_x_ decreased.

In the scenario where more cover crops were introduced alongside the EU‐wide 25% OA target, fertilizer inputs decreased similarly to the scenario without cover crops. However, SOC pools maintained the levels of the BAU scenario, while soil erosion and associated nutrient losses were significantly more mitigated. The P budget became slightly more negative due to increased crop and biomass exports. Conversely, the gross N budget and leaching increased substantially, surpassing both the scenarios without cover crops and the BAU scenario by 15.7%. This increase is attributed to enhanced N fixation from cover crops, which increased the total N inputs and led to a higher N surplus. Additionally, all gaseous N emissions in this scenario exceed BAU levels.

When the 25% of OA share was confined to individual member state borders, most nutrient fluxes and pools, such as SOC stock, soil erosion, and P budget, behaved similarly to the EU‐wide approach. However, a slight difference could be observed in the reduced nutrient inputs through fertilizers, which contributed to the slightly smaller N surplus, N leaching, and gaseous N emissions. Finally, incorporating cover crops did not significantly alter the stocks and pools compared to the EU‐wide selection.

### 3.4 Environmental Impact Comparison

**FIGURE 4:**
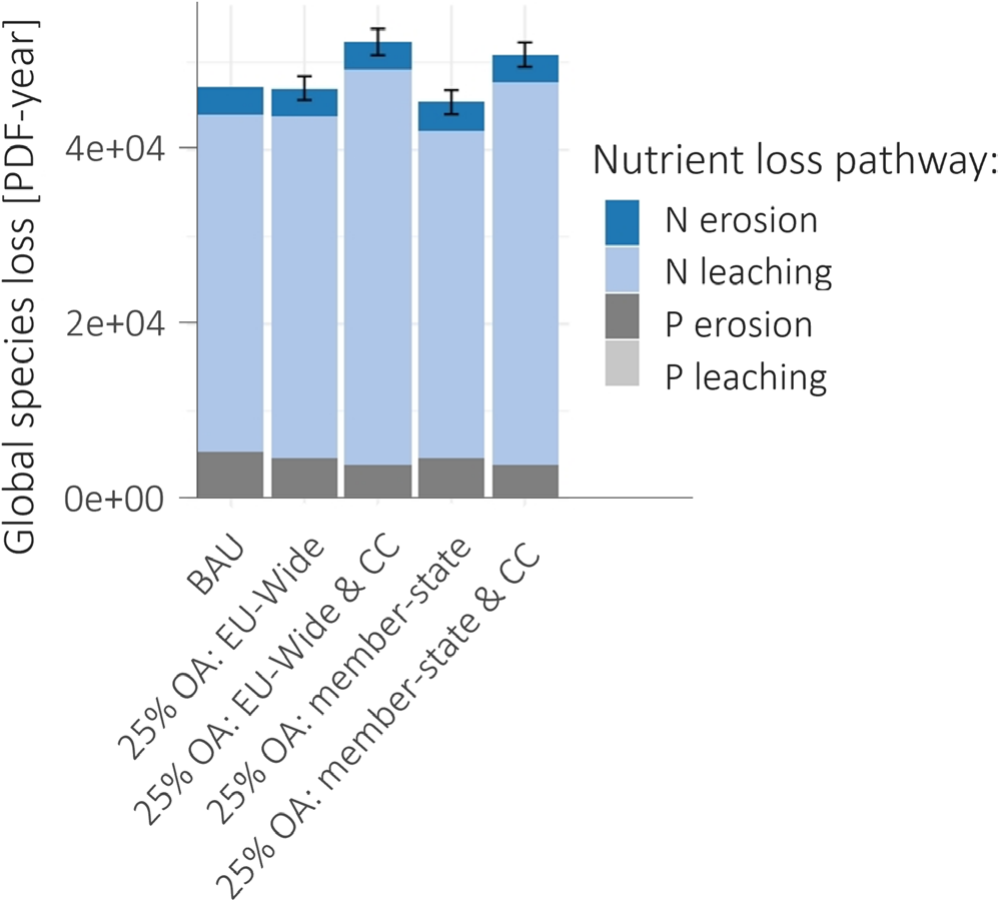
Average global species loss of BAU compared to 25% OA implemented EU‐wide or per member state, with or without additional cover crops (CC) during the crop rotation period from 2031 to 2034. The stacked bar plot details the impacts of each nutrient loss process. The error bar refers to the impact range of the low and high yield gap scenarios. A table presenting both average and marginal impacts can be seen in Table S9.

The BAU scenario contributed to freshwater eutrophication through P and N losses from agricultural soils via erosion and leaching, accounting for 4.72E‐04 PDF·year during the simulated crop rotation period from 2031 to 2034 (Figure 4 and Table S8), with N and P losses averaged over this 4‐year rotation period and average CFs.

Given the continuous duration of these losses, this translates to a potential loss of 0.05% of fish species due to freshwater eutrophication under BAU agricultural management in the EU countries. In comparison, Zhou et al. (2024) reported 10.7% global potential species loss when considering the N and P losses from agriculture across the world. Apart from the much smaller spatial extent, the lower impact of this study can also be attributed to the lower CFs found in Europe (due to lower effects on biodiversity) and the smaller P losses assumed in our study.

A clear hierarchy emerges in the contributions of processes to eutrophication impacts: N leaching > P erosion > N erosion > P leaching. N leaching accounts for the majority of potential species loss (77%), driven by the high N surplus, while P leaching contributed the least to the eutrophication impact. However, the impact of P should not be overlooked, particularly in Northern and Western Europe, where freshwater systems are predominantly P‐limited. This is exemplified by the fact that, although P erosion loads are smaller compared to those from N erosion, the environmental impacts of P erosion are higher. Among the 18 crop types analyzed, pulses, barley, and wheat exhibited the highest eutrophication impacts, likely due to their increased N inputs through biological fixation in pulses or their high dependency on N fertilizers.

When comparing eutrophication impacts, the scenario with 25% OA showed a slight reduction (−2.1%) compared to the BAU scenario, but only when using the member‐state approach. This reduction is attributed to decreased nutrient losses. When implemented EU‐wide, the impact remained essentially unchanged. This limited improvement is due to the selection criteria that identify many areas experiencing soil erosion rather than those with the most significant nutrient losses, such as areas with high N leaching. Implementing increased cover crops even raised the impact due to enhanced N fixation, which led to increased N leaching. The impacts derived from the marginal CFs indicated the same trend as when using the average CFs, highlighting the adverse effects of additional N loss, as seen in cover crop scenarios.

When applied EU‐wide, the impact remains largely unchanged. This limited improvement is primarily because the selected areas, while reducing soil erosion, did not target regions with the highest nutrient losses—areas where significant reductions could be achieved by focusing on high N leaching zones. Additionally, the inclusion of more cover crops actually exacerbated the impact due to increased N leaching. Analysis using marginal CFs confirmed this trend, indicating a detrimental effect with any additional N loss, particularly evident in the cover crop scenarios.

## 4 Discussion

### 4.1 Trade‐Offs and Synergies of Organic Agriculture Expansion

The expansion of OA to a 25% share in the EU and UK demonstrated its potential to considerably reduce dependency on mineral fertilizers and either lessen or maintain the current impact on biodiversity, depending on the changes in nutrient losses. However, this transition was accompanied by a significant trade‐off in yield reduction, widely documented in the literature (Barbieri et al. 2021; Seufert and Ramankutty 2017b; Seufert et al. 2012; Klinnert et al. 2024; Ponisio et al. 2015b). The model's regionalized assessment illustrated the influence of scaling OA across the EU as a whole or within individual member states. However, due to the abundance of degraded soil areas and the consistent application of selection criteria, the results did not vary substantially between these approaches. Nonetheless, the minor variations in N inputs and losses lessened the impact on freshwater ecosystems in the approach tailored to individual member states. Despite the challenges of achieving a 25% OA share, certain areas and regions showed promising potential for a transition that could lead to reduced nutrient losses while minimizing yield reductions, highlighting the nuanced outcomes of expanding OA.

The total reductions in grain and tuber yields of 9.7% to 11.4% combine a 4.4% decrease from the BAU scenario and a 5.5%–6.5% reduction from OA implementation, depending on the selection of target areas and the assumptions about the yield impact of plant protection measures. Notably, this observed decrease in grain and tuber production also reflects a shift in the crop rotation with increased fodder production. Despite differences in model and scenario assumptions, this yield decrease aligns with Bremmer et al. (2021), who reported a production decline of less than 10% assuming the Farm to Fork strategy implementation. The NPP experienced a smaller reduction of 4.9%–5.6%, and in some regions, even exceeded that of BAU. Therefore, expanding OA while sustaining grain and tuber production may require farmers to adjust their crop selections, possibly prioritizing other grains or tubers over fodder to strike a balance between production demands and environmental objectives. However, the additional fodder production in the EU and UK could offset current fodder imports from non‐EU countries, with our OA scenario replacing pulses and fodder imports entirely (Bruckner et al. 2019). Alternatively, with reduced livestock numbers, this fodder could be repurposed, either recycled back into the soil as green manure to increase SOC stock or used as feedstock to produce bio‐based products (Huo et al. 2024). In such scenarios, selecting areas to maximize NPP rather than grain and tuber yield would be more suitable.

However, the projected yield reduction should also be contextualized in light of current food waste, which constitutes 10% of the food supplied to EU consumers (Eurostat 2023), and the low energy efficiency of converting fodder into animal products in diets that are heavily reliant on these products (Sun et al. 2022). Therefore, reducing agricultural output may not necessitate increased imports or land use change if adjustments are made on the demand side, as suggested by Billen et al. (2021).

Incorporating the changed crop rotations into the scenario, which led to increased fodder production, is a strength of this study, as more complex and diversified rotations are a strategy for pest control in organic farming, while enhancing nutrient inputs through biological fixation (Barbieri et al. 2017). The inclusion of cover crops in the scenarios demonstrated the benefits of added N fixation, a crucial strategy for replacing mineral N fertilizers in OA. This approach not only increased C inputs into the soil, helping mitigate SOC decline and soil erosion, but also resulted in higher impacts on freshwater biodiversity due to increased N losses. However, N fixation increases by a factor of 3.6–7.8 in the cover crop scenario applied in all areas (i.e., 100% OA) may have overestimated the use of N‐fixing crops, compared to other studies reporting a 2.6‐fold increase (Barbieri et al. 2017). Therefore, careful assessment is needed to determine the appropriate amount of mineral N replacement when relying extensively on pulses, fodder crops, and N‐fixing cover crops to avoid exacerbating N surplus, with the option to substitute N‐fixing cover crops with non‐N‐fixing cover crops (Quemada et al. 2020). However, our extreme N‐fixing cover crop scenario suggests that the N supply in European organic agriculture may not be a limiting factor, provided the crop rotation incorporates a substantial proportion of N‐fixing crops and manure inputs stay constant.

Without N‐fixing cover crops, the 100% OA scenario resulted in a NPP production gap of 23.0% ± 1.3% compared to BAU, with the nutrient management responsible for 62.3%–77.4% of this gap. This finding aligns with other modeling studies and local observations that have highlighted the negative effect of N deficiency on crop production under large‐scale OA expansion (Barbieri et al. 2021; Gaudaré et al. 2023; Wijerathna‐Yapa et al. 2023). Similarly, Barbieri et al. (2021) found that N deficiency contributed 77% to the global cropland production gap, but with a higher overall production gap of 36% compared to our findings. This discrepancy stems from the different geographical contexts, with Europe being a nutrient‐importing region through global food and feed trade (Nesme et al. 2018). Additionally, our scenario maintained constant livestock numbers and included a larger N fixation than Barbieri et al.'s 20% reduction in livestock. Indeed, compared to Reimer et al. (2023), the N fixation of the 100% OA cover crop scenarios (54.0–55.8 kg N ha^−1^ year^−1^, corresponding to 57%–58% of total N inputs) falls within the range of 71 organic arable farms across 8 European regions (24–61 kg N ha^−1^ year^−1^, 33%–98% of total input).

The findings in this paper have reaffirmed the comprehensiveness of the DayCent model framework, known to include a large number of variables (Brilli et al. 2017) and its ability to interconnect the C, N, and P biogeochemical cycles. This integration enabled an evaluation not just of individual nutrient stocks or fluxes, but of critical elements that contribute to soil health and impacts on biodiversity (albeit only including one impact category). Evaluating these elements in isolation fails to capture the complete picture. For instance, in this study, transitioning all agricultural fields to OA resulted in a decrease in C input and SOC stock by 18.6% ± 1.4% and 0.9% ± 0.1% after 12 years, respectively; for comparison, a global study by Gaudaré et al. (2023) reports a 9% decline in SOC stocks over 20 years due to a 40% reduction in global soil C inputs. This indicates that their C input declines twice as much while their SOC loss is tenfold higher. This discrepancy can be explained by multiple factors beyond the different regions studied (global vs. EU) and the different durations (12 vs. 20 years): their study did not account for the yield gap caused by changes in plant protection measures nor any change in climate, which might have led to a higher C export than observed in our analysis. Additionally, they did not take the N‐C cycle interplay into account and, thus, might have overestimated the SOC stock reduction as SOC mineralization also releases N, which becomes available for plant uptake, potentially mitigating some of the SOC loss.

The impact assessment revealed that transitioning to OA in areas with N surplus could have led to a more substantial reduction in eutrophication impacts. Although the OA scenarios substantially reduced mineral fertilizer inputs and nutrient losses from erosion, these benefits only partially translated into the impact assessment as N leaching was the process with the highest impact. This emphasizes the importance of considering the regional conditions when evaluating agricultural management practices for maximum impact reduction. It also underscores the importance of conducting localized studies to develop effective, targeted mitigation strategies tailored to specific environmental and agricultural contexts.

### 4.2 Limitations and Uncertainties

Modeling the complex interactions between agricultural management and interconnected soil C‐, N‐, and P‐cycles offers benefits but requires appropriate assumptions and navigating the model's inherent limitations and uncertainties. We quantified the uncertainty related to the yield gap on the nutrient fluxes and stocks, but additional assumptions introduced further uncertainties. Firstly, we could have considered alternative nutrient management strategies in response to increasing OA or implemented more circular fertilization methods. Potential changes include shifts in livestock numbers (Muller et al. 2017), the limitation of conventional manure in organic farms (currently still permitted if on‐farm resources are insufficient (European Commission 2018)), alternative nutrient sources like sewage sludge (Billen et al. 2021; Cooper et al. 2018) and compost (Reimer et al. 2023; Martínez‐Blanco et al. 2013; Meyer‐Kohlstock et al. 2015), and increased use of manure for biogas production while increasing the nutrient availability (Burg et al. 2023). However, these changes might not be as prominent by 2034, the final year of the projection. Although approximately 50% of sewage sludge is currently applied to agricultural soils (Collivignarelli et al. 2019), this nutrient source was not accounted for in our study due to its relatively minor contribution, representing only about 2% of N and 6% of P inputs compared to mineral fertilizers (Huygens et al. 2022). Secondly, predicting future organic crop rotations presents challenges since current data cover limited areas and number of observations (53 organic and 46 conventional) and stem mainly from reported experimental field trials (Barbieri et al. 2017). Future policies and climate change might also influence the crop choice of the farmers. Additionally, yield gaps due to changes in plant protection measures remain uncertain, which is why we modeled a range of scenarios (10%–20% yield reduction) to capture this variability (see Section 2.1 in Supporting Information). Thirdly, other criteria, such as enhancing NPP or SOC stocks, could have guided the selection of areas to transition to OA, potentially altering our results significantly. Boosting SOC not only aids in climate change mitigation but also elevates soil health, reduces erosion, and bolsters crop yield and adaptability (De Rosa et al. 2024; Oldfield et al. 2019; Paustian et al. 2019).

Estimating the true benefits and drawbacks of increased OA remains challenging given the lack of information on (1) the distribution of the mineral fertilizer applications (and if rock P is still applied in organic farms across the EU), (2) the areas currently practicing OA, and, thus, (3) regions suitable for transitioning to OA. Enhancing our knowledge of these factors and integrating them into the model framework would strengthen the results. Nonetheless, both the baseline and OA scenarios are subject to the same uncertainties. Unlike other studies that focus on global scales and are therefore faced with a bias towards developed countries due to the data origins (Seufert et al. 2012; Barbieri et al. 2017; Ponisio et al. 2015b), this bias is less pronounced in this study since it focuses on the EU and UK. Missing flows should be included as soon as quality data is available for C (e.g., CH_4_ emissions), N (e.g., N input through seeds, sewage sludge application), and P (e.g., P input through seeds, atmospheric deposition, sewage sludge application) flows. Additionally, including missing land use types, such as permanent crops like orchards, vineyards, and olive groves, would further enhance the study's comprehensiveness.

Finally, additional uncertainties and limitations stem from the modeling framework itself or missing processes in DayCent, as discussed in Muntwyler et al. (2024), or the impact assessment method. For example, ammonia volatilization in DayCent is only indirectly represented, being linked to N in harvested or senesced biomass. It is thus included implicitly in the N surplus rather than simulated as a separate flux. We therefore report the gross N budget instead of incomplete fluxes, which likely results in an underestimation of this gaseous loss pathway, particularly in calcareous soils, and possible overestimation of other losses. The current model framework focuses on EU crop production, including nutrient fluxes and pools, but does not account for global supply chains (e.g., reduced yield may result in more imports from non‐EU countries), the demand side (e.g., dietary changes or food waste reduction) or assess the impact of the scenarios on categories other than freshwater eutrophication. Future studies could expand this model to compare the impacts of organic and conventional agriculture using additional LCA impact categories, such as climate change (including CO_2_, CH_4_, and N_2_O fluxes included in DayCent), providing a more comprehensive comparison of these systems. Also, soil erosion beyond rill‐ and inter‐rill erosion, such as erosion through tillage, gully, landslides, and wind, should be assessed further to identify differences in the agricultural management practices (Borrelli et al. 2023). While the model framework operates at high spatial resolution (1 km^2^), the inherent heterogeneity of soil conditions cannot fully capture very local conditions. The land use dataset used in this study extends to 2012 and may not fully reflect current land use. Future updates of the framework could incorporate more recent land use data (e.g., CORINE 2018) to improve temporal representativeness. Additionally, the CFs employed in the impact assessment are based on statistical relationships between nutrient levels and fish species richness but they do not account for direct exposure or damage caused by the nutrients to the fish (Morelli et al. 2018), nor do they consider other pressures that the fish species may face simultaneously or alternative biodiversity metrics such as functional diversity (Deksne et al. 2025).

## 5 Conclusion

Upscaling organic farming to 25% across EU and UK reduces total grain and tuber production by 6.4%–6.5% and slightly decreases the SOC pool, yet it simultaneously diminishes reliance on mineral fertilizer, nutrient surplus, and losses. The spatial specificity of our model enabled targeted interventions in areas previously degraded by management practices and fertilization strategies. By incorporating a detailed allocation procedure, the transition to organic agriculture could improve 0.8%–1.4% of degraded soils and lessen or maintain current impacts on freshwater fish due to eutrophication. However, uncertainties in our model's assumptions and input data mean outcomes should be interpreted at a regional rather than at a pixel level. Instead, regions highlighted as having a higher potential for transitioning to OA should be viewed as areas with promising prospects. These findings underscore the need for a comprehensive assessment of large‐scale agricultural management changes. The interconnected N, P, and C pools and fluxes should be systematically examined alongside crop yield, considering their respective environmental objectives and the potential for feedback mechanisms (e.g., excessive N fixation leading to increased N losses, reduced yields potentially inducing imports and land use change). This approach offers valuable insights into the synergies and trade‐offs between agricultural management practices and environmental consequences.

## Author Contributions

**Anna Muntwyler:** conceptualization, data curation, formal analysis, investigation, methodology, project administration, resources, software, validation, visualization, writing – original draft, writing – review and editing. **Emanuele Lugato:** conceptualization, data curation, methodology, resources, supervision, validation, writing – review and editing. **Panos Panagos:** conceptualization, data curation, supervision, validation, writing – review and editing. **Laura Scherer:** conceptualization, writing – review and editing. **Adrian Muller:** conceptualization, writing – review and editing. **Stephan Pfister:** conceptualization, data curation, funding acquisition, methodology, resources, software, supervision, validation, writing – review and editing.

## Conflicts of Interest

The authors declare no conflicts of interest.

## Supporting information


**Figure S1:** Flow chart adapted from Muntwyler et al. (2024) showing the data inputs, their spatial resolution.
**Figure S2:** Flow chart showing the selection criteria for the area to transition to organic agriculture.
**Figure S3:** Map showing the nutrient that limits crop growth in BAU or ORG in the period 2031–2034.
**Figure S4:** Biological N fixation (N_fix_), mineral N (N_min_), and mineral P (P_min_) inputs across scenarios.
**Figure S5:** Biological N fixation (N_fix_), mineral N (N_min_), and mineral P (P_min_) inputs across scenarios.
**Table S1:** Parameter descriptions of terms used in the main manuscript.
**Table S2:** Coefficients used to change the crop rotation based on the values for Europe in Barbieri et al.
**Table S3:** Current shares of OA based on EUROSTAT 2022 and % of agricultural area necessary to change.
**Table S4:** Carbon stocks and fluxes under the 100% OA scenario. The % of BAU is compared to current.
**Table S5:** Nitrogen stocks and fluxes under the 100% OA scenario. The % of BAU is compared to current.
**Table S6:** Phosphorus stocks and fluxes under the 100% OA scenario. The % of BAU is compared to current.
**Table S7:** EU average NPP, grain and tuber C production, and nutrient fluxes and stocks under the BAU.
**Table S8:** Global species loss of BAU compared to 25% OA implemented EU‐wide or per member.

## Data Availability

The manuscript includes full equations and parameters of the model in the text, the Supporting Information, or cited in publicly accessible literature. The data and maps will be available in the European Soil Data Centre (ESDAC) (Panagos, Van Liedekerke, et al. 2022) of the European Commission—Joint Research Centre: http://esdac.jrc.ec.europa.eu/.
