# Supplementary material for: Modeling Organic Agriculture Expansion in the EU: Assessing Productivity and Environmental Trade‐Offs
Source: Glob Chang Biol. 2026 Jul 13;32(7):e70973. doi: 10.1111/gcb.70973 (PMC13358403; doi:10.1111/gcb.70973)
Supplement: Supplementary file 1 — Figure S1: Flow chart adapted from Muntwyler et al. (2024) showing the data inputs, their spatial resolution. Figure S2: Flow chart showing the selection criteria for the area to transition to organic agriculture. Figure S3: Map showing the nutrient that limits crop growth in BAU or ORG in the period 2031–2034. Figure S4: Biological N fixation (Nfix), mineral N (Nmin), and mineral P (Pmin) inputs across scenarios. Figure S5: Biological N fixation (Nfix), mineral N (Nmin), and mineral P (Pmin) inputs across scenarios. Table S1: Parameter descriptions of terms used in the main manuscript. Table S2: Coefficients used to change the crop rotation based on the values for Europe in Barbieri et al. Table S3: Current shares of OA based on EUROSTAT 2022 and % of agricultural area necessary to change. Table S4: Carbon stocks and fluxes under the 100% OA scenario. The % of BAU is compared to current. Table S5: Nitrogen stocks and fluxes under the 100% OA scenario. The % of BAU is compared to current. Table S6: Phosphorus stocks and fluxes under the 100% OA scenario. The % of BAU is compared to current. Table S7: EU average NPP, grain and tuber C production, and nutrient fluxes and stocks under the BAU. Table S8: Global species loss of BAU compared to 25% OA implemented EU‐wide or per member. [file GCB-32-e70973-s001.pdf]

Supplementary Information for

Modelling Organic Agriculture Expansion in the EU: Assessing Productivity and Environmental Trade-offs

The Supporting Information includes further details on the following: (1) the model framework, (2) additional scenario assumptions, such as the coefficients for altering crop rotation and the current proportions of organic agriculture in the EU and UK, (3) the results of the factorial approach for transitioning 100% of the area to organic agriculture, and (4) supplementary findings referenced in the main text.

1. The model framework

In addition to the information provided in the methods section of the main text and the cited references, this section offers further details on the modelling framework to reduce the need for cross-referencing previously published studies. However, we have not reproduced all the details to maintain clarity and overview.

Plant production in DayCent is modelled as a crop-specific function influenced by genetic potential, phenology, nutrient availability, water and temperature stress, and solar radiation. This model dynamically allocates resources to various plant components, such as roots and shoots according to crop growth stage and environmental conditions. The C, N, and P submodels interact through different soil organic matter pools, which are regulated by CN and CP ratios, lignin content, and abiotic factors like water and temperature (Hartman et al., 2018). The model framework can investigate how agricultural management practices affect soil biogeochemistry. These practices include crop rotation (including cover crops), tillage, irrigation, plant harvest and residue management, and both organic and mineral fertilization. Figure S1 summarizes the data inputs, their spatial resolution, the model integration, and the model outputs; subsequent sub-sections provide further details on the modelling framework.

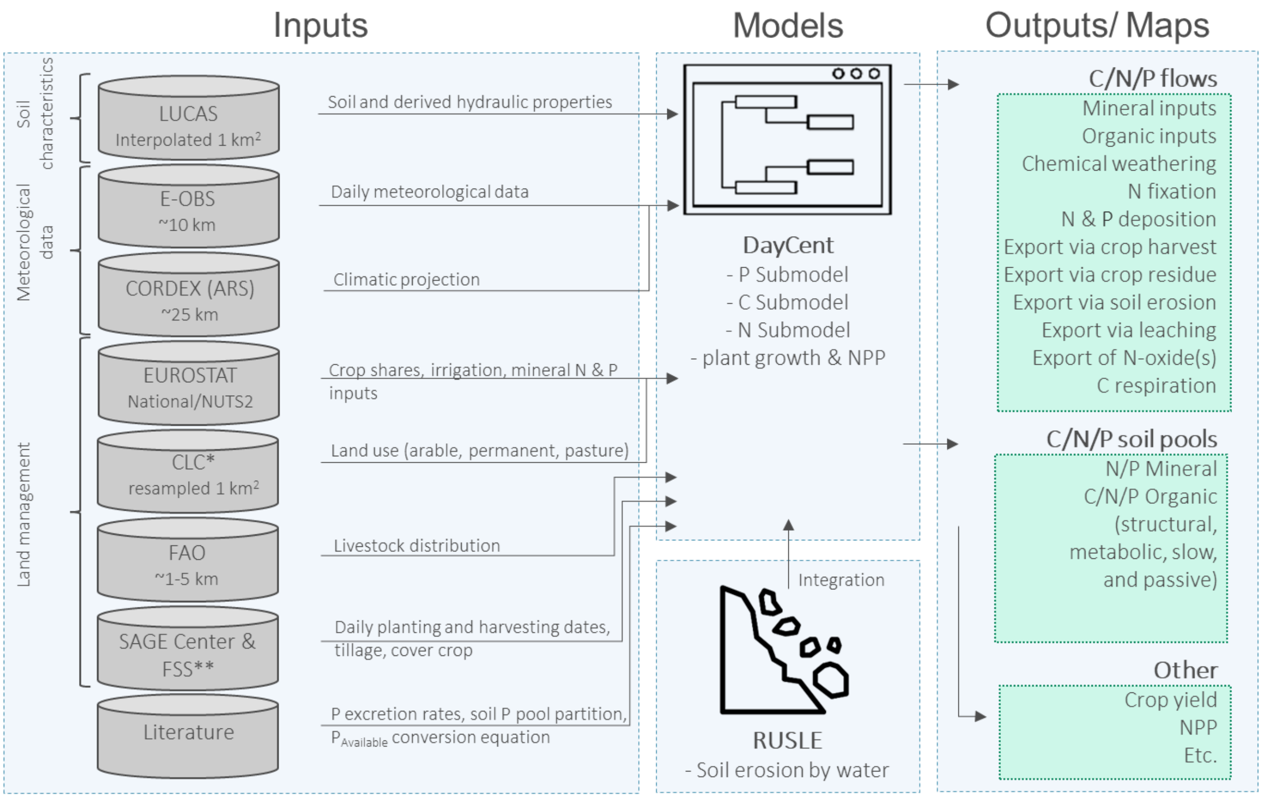

Figure S1 Flow chart adapted from Muntwyler et al. (2024) showing the data inputs, their spatial resolution, the model integration, and the model outputs. The inputs needed to run the DayCent model were derived from observationally derived datasets for soil characteristics and meteorological data, official statistics for land management practices, and calibrated values for the P submodel. Livestock excretion rates used to estimate the C, N, and P content coming from manure came from (Sattari, 2014; Sheldrick et al., 2003; Van Horn et al., 1996; Wilkerson et al., 1997) as described in (Lugato et al., 2014, 2017; Muntwyler et al., 2014) * Corine Land Cover, ** Farm Structure Survey

a. Land use and crop distribution

Land use and cover were derived from the Corine Land Cover (CLC) database to represent the main agricultural areas, classified into arable land, pasture, rice and heterogeneous agricultural zones. The crop distribution within arable land accounts for 18 major arable and fodder crop types, distributed at NUTS2: winter and spring barley, winter and spring wheat, forage and grain maize, soybean, sugar beet, sunflower, tobacco, ryegrass, alfalfa, rice, pulses, oilseed rape, cotton, and potato. Within each NUTS2 region, representative four-year crop rotations were developed and allocated across the simulated cropland area, based on their statistical (EUROSTAT) share. The crop rotation was defined by the four most cultivated crops within each NUTS2 region, permitting consecutive cultivation of the same crop when its share

exceeded 40% of the arable area. Details of the rotation ranking methods are described in (Lugato et al., 2014a).

Crop-specific parameterization (e.g., maximum potential growth rate *PRDX*, harvest index *HIMAX*, and CN ratios) followed Lugato et al. (2016, 2018b) and Pacifico et al., (2024), while P-related parameters (*PRAMX*, *PRAMN*) were taken from Muntwyler et al. (2023) when available. The spatial resolution of all land use and crop data was harmonized to 1 km², consistent with the DayCent framework.

a. Atmospheric N deposition and Weather data

Average wet and dry N deposition data were obtained from the European Monitoring and Evaluation Programme (EMEP) model (rv 4.5), aggregated to a 1 km² grid to align with the model resolution. We obtained daily maximum and minimum temperature and precipitation on a 10-km regular grid spatial resolution from the E-OBS gridded dataset (http://www.ecad.eu). The future climate projections from CNRM were bias-corrected to the historical E-OBS dataset, providing daily data of maximum and minimum temperature and precipitation on a grid of 0.22° resolution.

b. Crop calendars and managements

In the modelling framework, the crop production including its management was not changed from Lugato et al. (2016, 2018b) and Muntwyler et al. (2024). Planting and harvesting dates were derived from the global crop calendar dataset of the SAGE Center (https://nelson.wisc.edu/sage/data-and-models/crop-calendar-dataset/index.php). These defined the timing of sowing, harvest and, consequently, ploughing, irrigation, fertilizer and manure applications for each crop type and region were set according to agronomic principles. Conventional agro-techniques were assumed, including primary (mouldboard) and secondary tillage. Management practices (except mineral fertilization) were assumed to be constant across scenarios (e.g., ploughing was maintained in both conventional and organic systems due to the absence of EU-wide guidelines).

The following crops were simulated: winter and spring barley, durum and soft wheat, green (forage) and grain maize, and oilseed rape. Crop-specific residue retention was defined by parameters such as the fraction of aboveground residue removed. The Farm structure Survey (https://ec.europa.eu/eurostat/statistics-explained/index.php?title=Glossary:Farm_structure_survey_(FSS)), provided the application of reduced-tillage, no-tillage and cover crop adoption at NUT2 level for 2010 and 2016, which were implemented in the modelling framework accordingly. To estimate crop yields, the C in grain and tuber has to be converted

by using conversion factors (the C fraction of dry matter of that product which is approx. 50% and the moisture content of the product. In Pacifico et al. (2024), crop yields simulated with the biogeochemical DayCent model were evaluated against subnational yield data (2015–2018 averages) reported by Eurostat and national statistical institutes across the EU for soft wheat, barley, grain maize, and rapeseed. The study found that the DayCent framework successfully reproduces the yield levels of major crops and their spatial variability.

c. Mineral and organic nutrient input management

Mineral fertilizer inputs were derived from Eurostat (2013) decadal averages of national fertilizer consumption. Following the approach in Lugato et al. (2014, 2017), the national total fertilizer amount was allocated to each crop based on its theoretical agronomic N and P requirements, with preferential allocation to arable land (pasture received only 0–13% of total mineral P fertilizer). Each crop's N application was split into two events:

- 30% applied at planting, and
- 70% applied during the standing crop phase

P mineral application happened once per hear at planting. To account for spatial uncertainty, a probability density function (mean = 1, variance = 0.2) was applied to the mineral fertilization to generate variability in the inputs at the grid-cell level.

Organic nutrient inputs were derived from the FAO Gridded Livestock of the World dataset (Robinson et al., 2014). Livestock densities were converted into organic C, N, and P loads using species-specific excretion coefficients from Sattari (2014), Sheldrick et al. (2003), Van Horn et al. (1996), and Wilkerson et al. (1997). Manure was applied after harvest or during the growing season of high-demand crops (e.g., maize), constrained to a maximum rate of 170 kg N ha^{-1} yr^{-1}, following Nitrate Directive. The resulting maps of mineral and organic N and P inputs were produced at 1 km^2 resolution, ensuring spatial consistency with the DayCent simulations. They are available on the EUSO website.

The N fixation of added pulses in the crop rotation and the N-fixing cover crop was implemented by selecting the corresponding crop type, which includes a parameter defining the maximum potential N fixation rate (SNFXAC [g N fixed per g C fixed]; Hartmann et al., 2018). Consequently, N fixation is directly related to the plant growth rate. Symbiotic N fixation occurs only when mineral N is insufficient to meet

plant N demand, after accounting for all possible growth limitations, including P deficiency. The same cover crop was selected as in Muntwyler et al., (2024) and Lugato et al., (2018).

d. Soil model spin up

Table 1 Spin-up sequences simulated in each SCL unit

	Equil. 1	Equil. 2	*R*1	*R*2
Time	1700 yrs	300 yrs	1901–1960	1961–2010
Land use	3 yr (W-O-F) + pasture	4 yr (B-C-W-M) + pasture	Actual with more fodder crops in arable	Actual
Fertilization	Org	Org	Org + low Min	Org + Min
Tillage intensity	Low	Low	Moderate	Intensive
Irrigation	No	No	Yes	Yes

W-O-F = low yield wheat–oat –fallow ('maggese') rotation typical of roman and middle-age agriculture.
B-C-W-M = low yield barley–clover–wheat–meadow rotation introduced in XVII–XVIII century.
Org = organic fertilization; Min = mineral fertilization.

Taken from: Lugato, E., Panagos, P., Bampa, F., Jones, A., & Montanarella, L. (2014). A new baseline of organic carbon stock in European agricultural soils using a modelling approach. Global change biology, 20(1), 313-326.

Table S1. Parameter descriptions of terms used in the main manuscript

Parameter	Description
C_{Manure}	Input of C to the soil through manure
$C_{Org\ Leaching}$	Organic C leached from the soil organic layer into stream flow
$C_{Residue\ \&\ Roots\ Not\ Removed}$	Input of C to the soil from crop residues and crop roots left on the field
$C_{Soil\ Respiration}$	C respired during decomposition of SOC
HIMAX	Crop parameter for the maximum harvest index
N_2O	Nitrous oxide flux emitted from the soil
$N_{Crop\ Harvest}$	N removed from the system through crop harvest
$N_{Deposition}$	Input of N through atmospheric wet and dry N deposition
$N_{Fixation}$	Input of N through symbiotic and non-symbiotic N fixation
$N_{Leaching}$ (organic & mineral)	Organic or mineral N leached from the soil organic layer into stream flow
N_{Manure}	Input of N to the system through manure application
$N_{Mineral\ Fertilizer}$	Input of N to the soil through mineral fertilization
NO_x	Nitric oxide flux emitted from the soil
NPP	C production by grasslands and crops
$N_{Residue\ Removal}$	N removed through harvest of crop residues

Net $C_{Erosion}$	Net C transported to riverine system via soil erosion (water erosion through runoff, sheet, and rill erosion)
Net $N_{Erosion}$	Net N transported to riverine system via soil erosion (water erosion through runoff, sheet, and rill erosion)
Net $P_{Erosion}$	Net P transported to riverine system via soil erosion (water erosion through runoff, sheet, and rill erosion)
$P_{Crop\ Harvest}$	P removed from the system through crop harvest
$P_{Chemical\ Weathering}$	Input of P to the soil through weathering of parent material
$P_{Mineral\ Fertilizer}$	Input of P to the soil through mineral fertilization
P_{Manure}	Input of P to the system through manure application
$P_{Org\ Leaching}$	Organic P leached from the soil organic layer into stream flow
$P_{Residue\ Removal}$	P removed through harvest of crop residues
PRDX	Crop parameter for the maximum potential crop productivity

2. Additional scenario assumptions

2.1. Yield gap considerations

Our approach separates the yield gap components that DayCent can model mechanistically (nitrogen availability, crop rotation effects) from those it cannot (pest, disease, and weed pressure). Several lines of evidence support including a plant-protection-related yield gap for assessing organic agriculture:

A. YIELD GAP WITH SIMILAR NITROGEN INPUTS

Studies show yield gaps in organic systems even when nitrogen inputs are similar or higher than in conventional systems:

- Ponisio et al. (2015) found a yield gap (9+4%) where organic systems received more or similar N than conventional ones. When N inputs were higher in organic treatments, the yield gap was intermediate and more variable (17+6%), and significantly different from the yield ratio with similar N input (figure 2). “The influence of (c) nitrogen input on the organic-to-conventional yield ratio”:

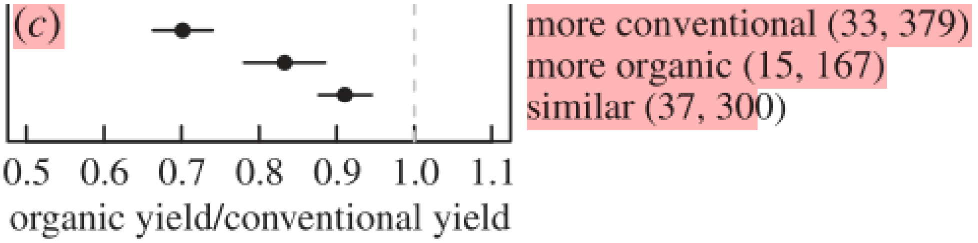

- Seufert et al. (2012) found significantly larger yield gaps when levels of nitrogen input were similar or lower in the organic to conventional treatments, compared with cases where nitrogen input was higher in the organic treatment. In all cases, a yield gap was found. Influence of N input on organic-to-conventional yield ratios:

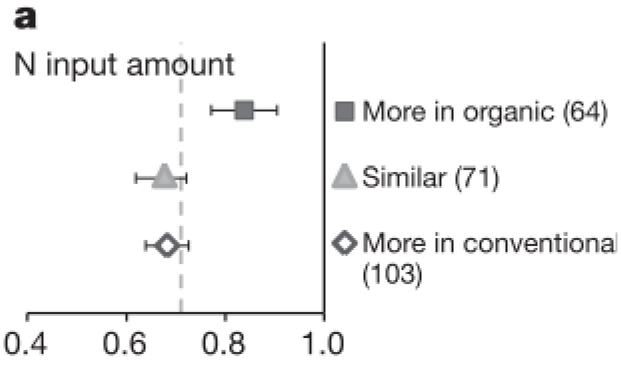

- This variability in the literature regarding non-N-related yield gaps motivated our decision to model a range of yield gap scenarios.

B. LEGUME YIELD GAP

Even nitrogen-fixing legumes (which depend less on external nitrogen inputs) show yield gaps in organic systems (Seufert et al., 2019; Ponisio et al., 2015; De Ponti et al., 2012; Seufert et al., 2012), suggesting factors beyond N limitation contribute to yield differences.

- Seufert et al. 2019: Yield gap of soybean:

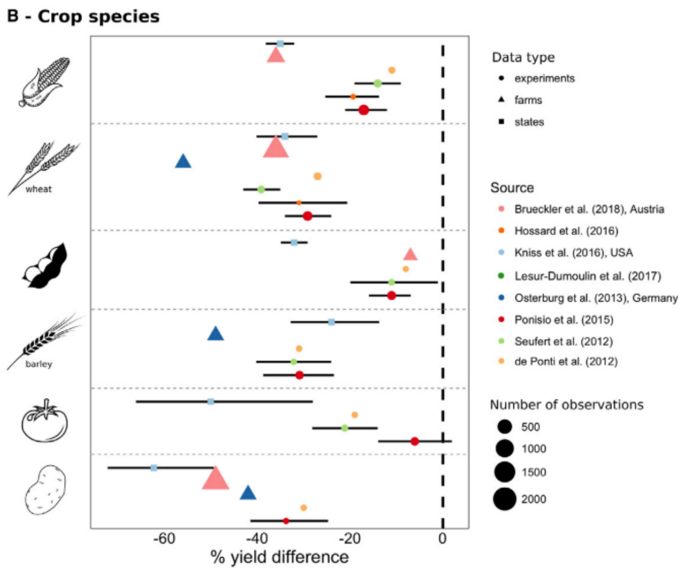

- Ponisio et al., 2015: The organic-to-conventional yield ratio of legumes:

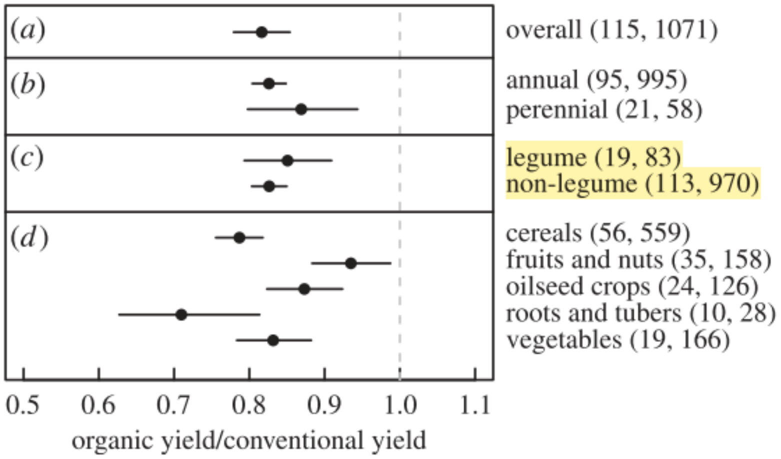

- Ponti et al. 2012: averages and ranges of the organic-conventional relative yields:

Table 1
Number of data entries, averages and ranges of the organic–conventional relative yields of selected crop groups and crops. Averages of crop groups follo common letter were not significantly different according to the test for multiple comparisons of Bonferroni ($P < 0.05$). Within each crop group crops are listec yield.

Crop	n[a]	Relative yield Average (%)	Relative yield Range (%)	Remarks
Cereals	156	79 ab	40–145	
Rice (*Oryza sativa* L.)	7	94	86–105	
Corn (*Zea mays* L.)	34	89	60–141	Almost all in North-America (26); some other countries (8)
Oats (*Avena sativa* L.)	14	85	40–145	–
Other cereals	8	81	45–111	Triticale (3), unspecified cereals (3), buckwheat (1), sorghum (1)
Rye (*Secale cereale* L.)	7	76	63–104	Data comprise spring, fall, and winter rye
Wheat (*Triticum* spp. L.)	66	73	40–130	Data comprise spring, summer, winter, and durum wheat
Barley (*Hordeum vulgare* L.)	20	69	46–105	Data comprise spring, summer, and winter barley
Root and tuber crops	24	74 a	37–114	
Potato (*Solanum tuberosum* L.)	21	70	37–114	All from European countries
Other root and tuber crops	3	105	89–114	Sweet potato (2), sugar beets (1)
Pulses	39	88 b	48–126	
Soybean (*Glycine max* L.)	16	92	74–126	Virtually all from USA (14)
Other pulses	12	91	67–121	Green beans (4), and other pulses.
Pea (*Pisum sativum* L.)	9	85	67–100	

- Seufert et al. 2012: Influence of plant type on organic-to-conventional yield ratios:

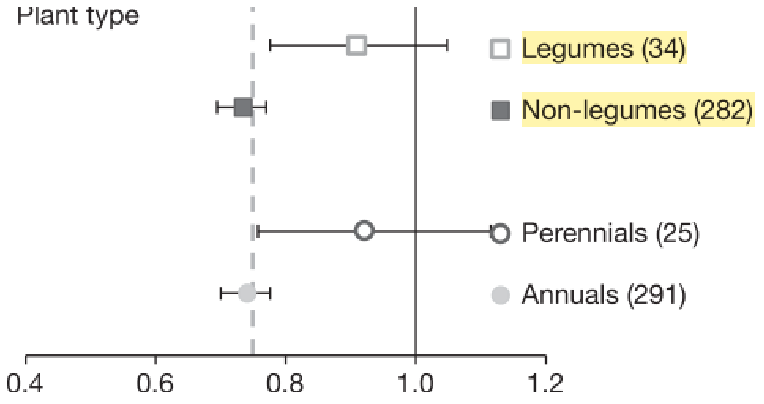

C. PRECEDENT IN SIMILAR STUDIES

Other researchers modeling organic agriculture expansion have included additional yield gaps to account for factors beyond nitrogen and crop rotation:

- Barbieri et al. (2021): “We corrected this organic-to-conventional yield gap to account for yield reduction due to pests, diseases and weeds only (ranging from 0 to 18%, depending on the crop species), by not accounting for the yield reduction due to N deficiency. This is because the GOANIM model itself simulates the yield reduction due to N limitation.“

D. PESTICIDE-SPECIFIC YIELD IMPACTS:

Recent research on natural pest control specifically examining pesticide effects shows measurable impacts:

- Klinnert et al. (2024): "The average regional yield gap in the EU between organic and conventional farming attributable to pesticides use from our estimations is −15%, with important differences associated to crops, ranging between −8% in legumes, −17% in barley and −23% in rye."

To approximate this organic-to-conventional yield reduction of 20% and 10%, the maximum potential crop productivity in the model was decreased by 40% (i.e., high yield gap) and 20%, respectively. This adjustment was needed, as actual yields are also influenced by multiple limiting factors such as climate, and nutrient and water availability (Hartman et al., 2018). Therefore, lowering the maximum potential productivity also reduces these limitations (e.g., lower N and water demand), resulting in a smaller yield reduction than the decrease set for the maximum potential productivity.

2.2. Coefficients

Table S2. Coefficients used to change the crop rotation based on the values for Europe in Barbieri et al. (2019). The ratios above 1 mean that the harvested area increased when switching to organic agricultural management for this crop category. On the contrary, ratios below 1 decreased the share of the crop in the crop rotation

Crop category	Organic-to conventional ratio of timeshare in rotations
Primary cereals	0.705
Secondary cereals	0.838
Pulses	1.398
Oil crops	0.266
Root crops	0.617
Temporary fodders	1.602
Industrial crops	0.651

Table S3. Current shares of OA based on EUROSTAT 2022 and % of agricultural area necessary to change. In 2019, Austria had already reached the 25% organic production target. In contrast, Malta (0.62%), Ireland (2.2%), Bulgaria (2.2%), and the UK (2.62%) have currently low shares and need substantial expansion to meet the 25% target. Yet, in terms of absolute areas, countries with the largest agricultural areas, such as France, Germany, and Poland, also have the largest areas to transition.

NUTS0	Total fully converted and under conversion to organic farming	Share necessary	Area necessary	EUROSTAT notes
	%	%	ha	
AT	25.69	0.00	0	data from 2021
BE	7.60	17.40	253848.6	
BG	2.20	22.80	995790	
CY	6.30	18.70	61934.4	provisional
CZ	15.96	9.04	341974.2	
DE	9.83	15.17	2872925	
DK	11.43	13.57	371166.6	
EE	23.42	1.58	15517.18	
EL	17.22	7.78	205781	
ES	10.83	14.17	1804819	
FI	14.98	10.02	22158304	
FR	10.06	14.94	129749	estimated
HR	8.94	16.06	4403431	
HU	6.31	18.69	262597.1	break
IE	2.20	22.80	1075310	
IT	18.14	6.86	884343.6	
LT	9.32	15.68	710778.3	
LU	6.23	18.77	523225.9	
LV	15.88	9.12	17850.27	
MT	0.62	24.38	187717	
NL	4.44	20.56	73.14	
PL	3.91	21.09	459865.5	
PT	19.31	5.69	3619592	estimated

RO	5.08	19.92	103688.9	
SE	19.94	5.06	2103672	
SI	11.10	13.90	154972.6	
SK	13.69	11.31	58616.3	estimated
UK	2.62	22.38	211327.4	data from 2019

2.3. Area selection approach details

The area was selected in sequence: first, areas exceeding one or more soil health thresholds, and secondly, areas exhibiting the smallest potential yield reductions when converted to OA (Fig. SI 2). The EU-wide approach selected areas within degraded areas with the lowest yield reduction across all member states simultaneously. In the member-state approach, some countries faced limitations in meeting the selection criteria. Ireland, Latvia, and Lithuania lacked sufficient areas that both exceeded the soil health thresholds and produced grain or tubers to cover the target area. Ireland requires substantial areas to convert to organic agriculture to meet the 25% target but predominantly consists of grass- and fodder-based agriculture that lacks grain or tuber crops. Conversely, Latvia and Lithuania had few areas identified as degraded. Consequently, in these cases, the selection was based on net primary productivity (NPP) rather than crop yield.

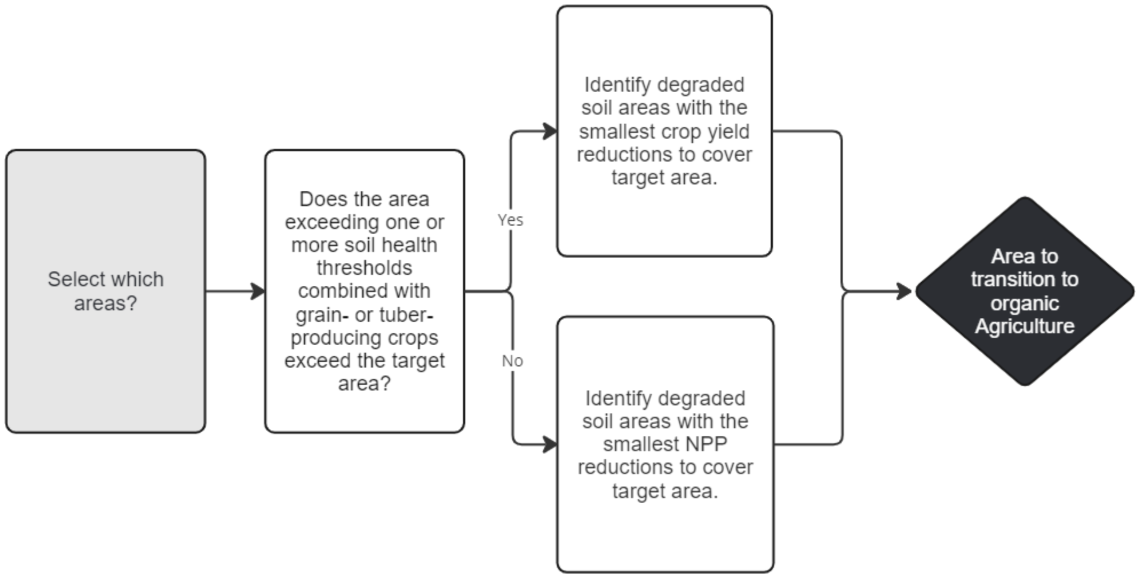

Figure S2. Flow chart showing the selection criteria for the area to transition to organic agriculture. The same rules were applied either per member state or EU-wide.

3. Results

3.1. Factorial analysis of 100% organic agriculture

Table S4. Carbon stocks and fluxes under the 100% OA scenario. The % of BAU is compared to current. The % of the scenarios is compared to BAU.

	NPP	C in grain & tuber	C export[b]	Net C erosion	Net soil erosion	C budget[a]	SOC
	[kg ha^{-1} $year^{-1}$]	[kg ha^{-1} $year^{-1}$]	[kg ha^{-1} $year^{-1}$]	[kg ha^{-1} $year^{-1}$]	[kg ha^{-1} $year^{-1}$]	[kg ha^{-1} $year^{-1}$]	[kg ha^{-1}]
Current	6767.0	1518.4	3.27	8.79	423.0	3.36	78.5
BAU	6582.6 (97.2%)	1452.2 (95.6%)	3.16 (96.5%)	8.67 (98.6%)	422.6 (95.63%)	3.28 (97.3%)	78.1 (99.5%)
BAU_noMin	4724.1 (71.7%)	825.92 (56.8%)	2.03 (64.4%)	8.48 (97.8%)	420.6 (56.87%)	2.61 (79.6%)	76.9 (98.4%)
ORG	6738.3 (102.%)	1172.8 (80.7%)	3.07 (97.1%)	7.93 (91.4%)	378.4 (80.76%)	3.30 (100.7%)	78.6 (100.6%)
ORG_noMin	5451.4 (82.8%)	765.65 (52.7%)	2.29 (72.4%)	7.81 (90.1%)	377.2 (52.72%)	2.86 (87.3%)	77.8 (99.5%)
ORG_noMin Yield gap 10%	5158.8 (78.3%)	676.81 (46.6%)	2.14 (67.9%)	7.77 (89.5%)	376.7 (46.60%)	2.71 (82.7%)	77.5 (99.1%)
ORG_noMin Yield gap 20%	4981.2 (75.6%)	610.85 (42.0%)	2.06 (65.2%)	7.75 (89.3%)	376.6 (42.06%)	2.62 (79.9%)	77.3 (98.9%)
ORG_noMin Yield gap 10% & CC	7162.2 (108.%)	906.81 (62.4%)	2.54 (80.4%)	7.41 (85.4%)	336.6 (62.44%)	4.12 (125.8%)	79.6 (101.9%)
ORG_noMin Yield gap 20% & CC	6877.7 (104.%)	783.54 (53.9%)	2.38 (75.3%)	7.39 (85.1%)	336.5 (53.95%)	4.00 (122.1%)	79.4 (101.6%)

[a] corresponding to the C budget defined in Equation (1)

[b] corresponding to the terms exporting C in Equation (1): $C_{Soil\ Respiration} + C_{Org\ Leaching} + \text{Net } C_{Erosion}$

Table S5. Nitrogen stocks and fluxes under the 100% OA scenario. The % of BAU is compared to current. The % of the scenarios is compared to BAU.

	Net N erosion	Mineral N input	Organic N input	N fixation	Total N input	Gross N budget[a]	N export[b]	Nmin leaching	Norg leaching	N deposition	N2O-N	Total soil N
	[kg ha^{-1} $year^{-1}$]	[kg ha^{-1} $year^{-1}$]	[kg ha^{-1} $year^{-1}$]	[kg ha^{-1} $year^{-1}$]	[kg ha^{-1} $year^{-1}$]	[kg ha^{-1} $year^{-1}$]	[kg ha^{-1} $year^{-1}$]	[kg ha^{-1} $year^{-1}$]	[kg ha^{-1} $year^{-1}$]	[kg ha^{-1} $year^{-1}$]	[kg ha^{-1} $year^{-1}$]	[kg ha^{-1}]
Current	0.95	57.7	40.2	15.2	113.1	43.4	80.3	33.9	0.51	10.6	2.25	8477.6
BAU	0.94 (98.8%)	57.7 (100%)	40.2 (100%)	15.1 (99.8%)	113.1 (99.9%)	45.8 (105.6%)	77.8 (96.9%)	34.8 (102.6%)	0.49 (96.2%)	10.6 (100.%)	2.32 (103.1%)	8443.2 (99.5%)
BAU_noMin	0.92 (97.8%)	0 (0%)	40.2 (100%)	14.2 (94.2%)	54.49 (48.1%)	15.0 (32.9%)	50.0 (64.2%)	20.3 (58.4%)	0.33 (67.6%)	10.6 (100.%)	1.50 (64.6%)	8293.4 (98.2%)
ORG	0.86 (91.3%)	48.3 (83.6%)	40.2 (100%)	62.1 (409.4%)	150.6 (133.2%)	81.3 (177.4%)	79.9 (102.6%)	58.8 (168.9%)	0.49 (99.9%)	10.6 (99.9%)	2.71 (116.6%)	8506.0 (100.7%)
ORG_noMin	0.84 (89.7%)	0 (0%)	40.2 (100%)	57.6 (379.8%)	97.85 (86.5%)	47.9 (104.4%)	60.5 (77.7%)	39.9 (114.6%)	0.36 (73.1%)	10.6 (99.9%)	1.90 (82.0%)	8383.9 (99.2%)
ORG_noMin Yield gap 10%	0.84 (89.4%)	0 (0%)	40.2 (100%)	55.8 (368.2%)	96.09 (84.9%)	48.5 (105.9%)	58.1 (74.6%)	42.1 (120.8%)	0.35 (71.1%)	10.6 (99.9%)	1.91 (82.3%)	8364.2 (99.0%)

ORG_noMin Yield gap 20%	0.84 (89.2%)	0 (0%)	40.2 (100%)	54.0 (356.0%)	94.23 (83.3%)	48.5 (105.9%)	56.2 (72.2%)	43.3 (124.4%)	0.34 (69.8%)	10.6 (99.9%)	1.90 (82.1%)	8350.5 (98.9%)
ORG_noMin Yield gap 10% & CC	0.80 (84.8%)	0 (0%)	40.4 (100.7%)	118. (783.2%)	159.3 (140.8%)	102. (223.1%)	67.6 (86.8%)	68.6 (196.8%)	0.63 (128.%)	10.6 (99.9%)	3.32 (143.1%)	8600.3 (101.8%)
ORG_noMin Yield gap 20% & CC	0.80 (84.6%)	0 (0%)	40.4 (100.7%)	118. (779.1%)	158.7 (140.3%)	105. (229.5%)	64.0 (82.2%)	43.3 (124.4%)	0.34 (69.8%)	10.6 (99.9%)	3.36 (144.7%)	8583.7 (101.6%)

a) corresponding to the N budget defined in Equation (3)

b) corresponding to the terms exporting N in equation (3): $N_{Crop\ Harvest} + N_{Residue\ Removal}$

Table S6. Phosphorus stocks and fluxes under the 100% OA scenario. The % of BAU is compared to current. The % of the scenarios is compared to BAU.

	Net P erosion	Mineral P input	Organic P input	Total P input	P export c)	P budget b)	Total soil P	Available soil P a)	P leaching
	[kg ha^{-1} $year^{-1}$]	[kg ha^{-1} $year^{-1}$]	[kg ha^{-1} $year^{-1}$]	[kg ha^{-1} $year^{-1}$]	[kg ha^{-1} $year^{-1}$]	[kg ha^{-1} $year^{-1}$]	[kg ha^{-1}]	[kg ha^{-1}]	[kg ha^{-1} $year^{-1}$]
Current	0.24	6.35	6.52	12.88	12.91	-0.28	2240.1	727.0	0.00
BAU	0.24 (100%)	6.35 (99.9%)	6.52 (100%)	12.88 (100%)	12.36 (95.6%)	0.27 (-99.0%)	2246.4 (100.2%)	714.2 (98.2%)	0.00 (94.8%)
BAU_noMin	0.23 (97.9%)	0 (0%)	6.52 (100%)	6.52 (50.6%)	8.12 (65.7%)	-1.84 (-665.4%)	2217.4 (98.7%)	670.0 (93.8%)	0.00 (83.7%)
ORG	0.21 (89.1%)	5.23 (82.3%)	6.52 (99.9%)	11.76 (91.3%)	11.89 (96.1%)	-0.35 (-124.9%)	2233.1 (99.4%)	671.2 (93.9%)	0.00 (93.7%)
ORG_noMin	0.21 (87.4%)	0 (0%)	6.52 (99.9%)	6.52 (50.6%)	8.97 (72.6%)	-2.67 (-963.0%)	2204.9 (98.1%)	618.6 (86.6%)	0.00 (83.2%)
ORG_noMin Yield gap 10%	0.21 (87.6%)	0 (0%)	6.52 (99.9%)	6.52 (50.6%)	8.48 (68.6%)	-2.18 (-785.3%)	2210.9 (98.4%)	644.1 (90.1%)	0.00 (79.8%)
ORG_noMin Yield gap 20%	0.21 (87.7%)	0 (0%)	6.52 (99.9%)	6.52 (50.6%)	8.12 (65.7%)	-1.82 (-656.1%)	2215.4 (98.6%)	661.5 (92.6%)	0.00 (78.0%)
ORG_noMin Yield gap 10% & CC	0.19 (77.6%)	0 (0%)	6.56 (100.5%)	6.56 (50.9%)	9.63 (77.9%)	-3.27 (-1179.%)	2199.2 (97.8%)	661.5 (92.6%)	0.00 (111.%)
ORG_noMin Yield gap 20% & CC	0.19 (77.7%)	0 (0%)	6.56 (100.5%)	6.56 (50.9%)	9.01 (72.9%)	-2.64 (-954.5%)	2205.8 (98.1%)	591.3 (82.7%)	0.00 (109.%)

a) Measured in Olsen-P

b) corresponding to the P budget defined in Equation (2)

c) corresponding to the terms exporting P in equation (2): $P_{Crop\ Harvest} + P_{Residue\ Removal} + Net\ P_{Erosion} + P_{Org\ Leaching}$

Table S7. EU average NPP, grain and tuber C production, and nutrient fluxes and stocks under the BAU scenario and 25% organic agriculture implementation, both EU-wide and by member state, over the whole crop rotation. Percentage values show deviations from the BAU scenario. In this table, the values are the averages of both low/high yield gap scenarios ± with the ranges of the two applied yield gaps (only from plant protection measures). Mt equals Tg.

Variable	Unit	BAU	EU-Wide	EU-Wide incl. cc	Member-state	Member-state incl. cc
NPP C	t ha^{-1} $year^{-1}$	6.58	6.41±0.03	6.58±0.0	6.41±0.03	6.58±0.0
			(-2.6%)	(-0.0%)	(-2.6%)	(-0.0%)
NPP C	Mt $year^{-1}$	1039.1	1011.6±3.5	1039.1±0.0	1012.0±3.9	1039.1±0.0
			(-2.6%)	(-0%)	(-2.6%)	(-0%)
C grain & tuber	t ha^{-1} $year^{-1}$	1.45	1.36±0.02	1.37±0.02	1.36±0.02	1.37±0.02
			(-6.4%)	(-5.7%)	(-6.5%)	(-5.5%)
C grain & tuber	Mt $year^{-1}$	229.2	214.6±1.7	216.1±1.8	214.2±1.7	216.7±2.0
			(-6.4).	(-5.7)	(-6.5%)	(-5.5%)
SOC	t ha^{-1} $year^{-1}$	78.16	78.06±0.02	78.16±0.0	78.07±0.03	78.16±0.0
			(-0.1%)	(-0.0%)	(-0.1%)	(-0.0%)
C input	t ha^{-1} $year^{-1}$	3.28	3.22±0.02	3.39±0.01	3.22±0.02	3.4±0.01
			(-1.9%)	(+3.3%)	(-1.9%)	(+3.6%)
Net soil erosion	t ha^{-1} $year^{-1}$	0.42	0.415±<0.01	0.405±<0.01	0.417±<0.01	0.410±<0.01
			(-1.9%)	(-4.2%)	(-1.5%)	(-3.1%)
Net C erosion	kg ha^{-1} $year^{-1}$	8.68	8.52±0.02	8.41±0.04	8.57±0.01	8.49±0.02
			(-1.8%)	(-3.1%)	(-1.3%)	(-2.1%)
C respiration	kg ha^{-1} $year^{-1}$	3.28	3.22±0.02	3.38±0.01	3.23±0.02	3.4±0.01
			(-1.8%)	(+3.1%)	(-1.7%)	(+3.6%)
C budget	kg ha^{-1} $year^{-1}$	-15.06	-20.57±1.33	-9.54±1.56	-20.06±1.56	-14.55±1.07
			(+36.6%)	(-36.6%)	(+33.2%)	(-3.4%)
Soil P total	kg ha^{-1} $year^{-1}$	2246.5	2240.76±0.96	2239.82±1	2240.6±0.95	2239.35±1.07
			(-0.3%)	(-0.3%)	(-0.3%)	(-0.3%)
Soil P available	kg ha^{-1} $year^{-1}$	714.2	701.69±3.69	693.25±3.72	701±3.62	691.49±3.91
			(-1.8%)	(-2.9%)	(-1.9%)	(-3.2%)
P budget	kg ha^{-1} $year^{-1}$	0.28	-0.15±0.08	-0.19±0.09	-0.16±0.09	-0.22±0.1
			(-154.7%)	(-168.3%)	(-158.2%)	(-178.9%)

P leaching	kg ha^{-1} year^{-1}	0.005	0.0047±<0.001 (-2.3%)	0.0048±<0.001 (+0.7%)	0.0047±<0.001 (-2.2%)	0.0048±<0.001 (+1.1%)
Mineral P input	kg ha^{-1} year^{-1}	6.36	5.34±0.01 (-16.0%)	5.35±0.01 (-15.8%)	5.35±0.0 (-15.8%)	5.36±0.0 (-15.6%)
Mineral P input	t year^{-1}	10048800	8437200 (-16.0%)	8453000 (-15.8%)	8437200 (-15.8%)	8468800 (-15.6%)
Mineral P saved	t year^{-1}	0	1611600	1595800	1611600	1580000
Net P erosion	kg ha^{-1} year^{-1}	0.24	0.24±<0.01 (-2.3%)	0.23±<0.01 (-4.7%)	0.24±<0.01 (-1.7%)	0.24±<0.01 (-3.4%)
Soil N total	kg ha^{-1} year^{-1}	8443.3	8432.0±1.8 (-0.1%)	8460.6±0.78 (+0.2%)	8431.1±2.26 (-0.1%)	8460.6±1.62 (+0.2%)
N budget	kg ha^{-1} year^{-1}	45.86	44.62±0.22 (-2.7%)	53.06±0.68 (+15.7%)	44.48±0.0 (-3.0%)	52.7±0.41 (+14.9%)
Mineral N input	kg ha^{-1} year^{-1}	57.72	48.92±0.02 (-15.2%)	49.0±0.03 (-15.1%)	48.66±0.13 (-15.7%)	48.76±0.13 (-15.5%)
Mineral N input	t year^{-1}	91197600	77293600 (-15.2%)	77420000 (-15.1%)	76882800 (-15.7%)	77040800 (-15.5%)
Mineral N saved	t year^{-1}	0	13904000	13777600	14314800	14156800
N fixation	kg ha^{-1} year^{-1}	15.17	20.18±0.16 (+33.0%)	29.11±0.27 (+91.8%)	20.31±0.27 (+33.9%)	29.18±0.04 (+92.3%)
N leaching	kg ha^{-1} year^{-1}	35.35	35.2±0.27 (-0.4%)	39.0±0.64 (+10.3%)	34.94±0.14 (-1.1%)	38.81±0.46 (+9.8%)
N_2O-N	kg ha^{-1} year^{-1}	2.32	2.25±0.0 (-3.3%)	2.48±0.02 (+6.6%)	2.24±0.0 (-3.6%)	2.45±0.01 (+5.3%)
NO_x-N	kg ha^{-1} year^{-1}	1.93	1.88±<0.01 (-2.3%)	1.99±<0.01 (+3.1%)	1.87±<0.01 (-3.3%)	1.99±<0.01 (+3.0%)
N_2	kg ha^{-1} year^{-1}	3.73	4.07 (+9.3%)	4.75 (+27.4%)	4.08 (+9.5%)	4.53 (+21.6%)
Net N erosion	kg ha^{-1} year^{-1}	0.95	0.93±<0.01 (-1.8%)	0.92±<0.01 (-3.1%)	0.93±<0.01 (-1.3%)	0.93±<0.01 (-2.2%)

3.2. Additional results

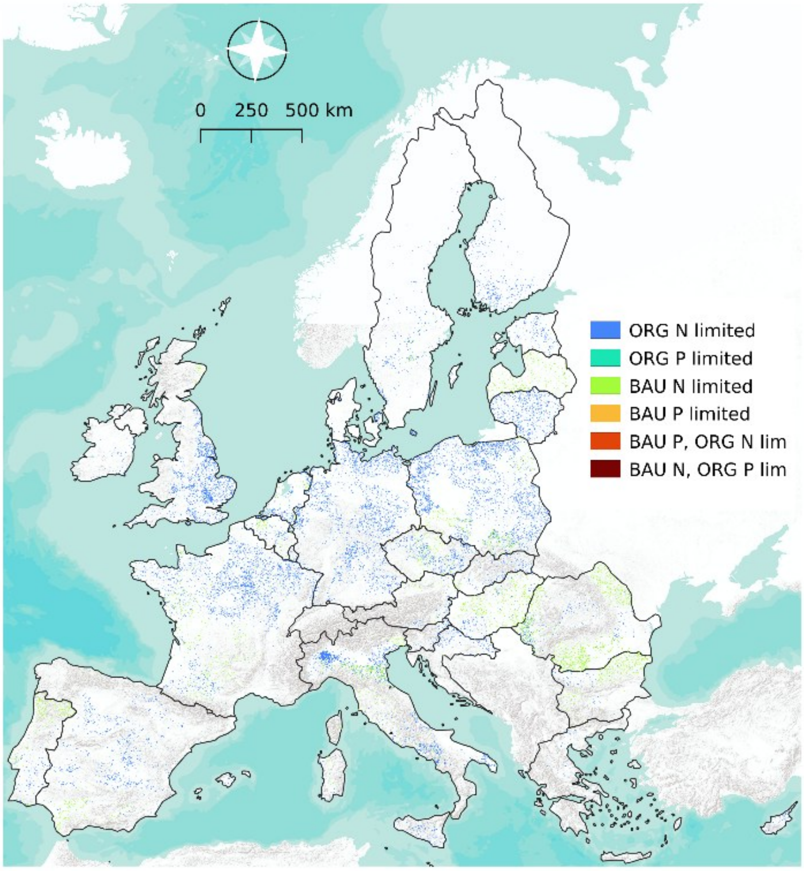

Figure S3. Map showing the nutrient that limits crop growth in BAU or ORG in the period 2031-2034.

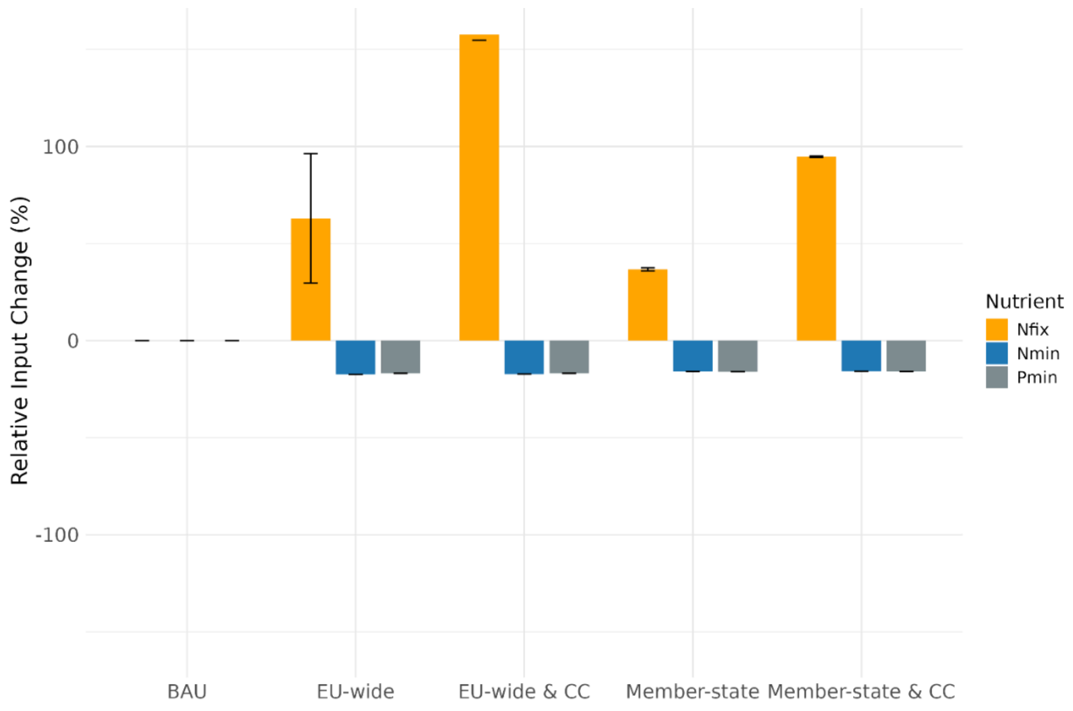

Figure S4. Biological N fixation (Nfix), mineral N (Nmin), and mineral P (Pmin) inputs across scenarios.

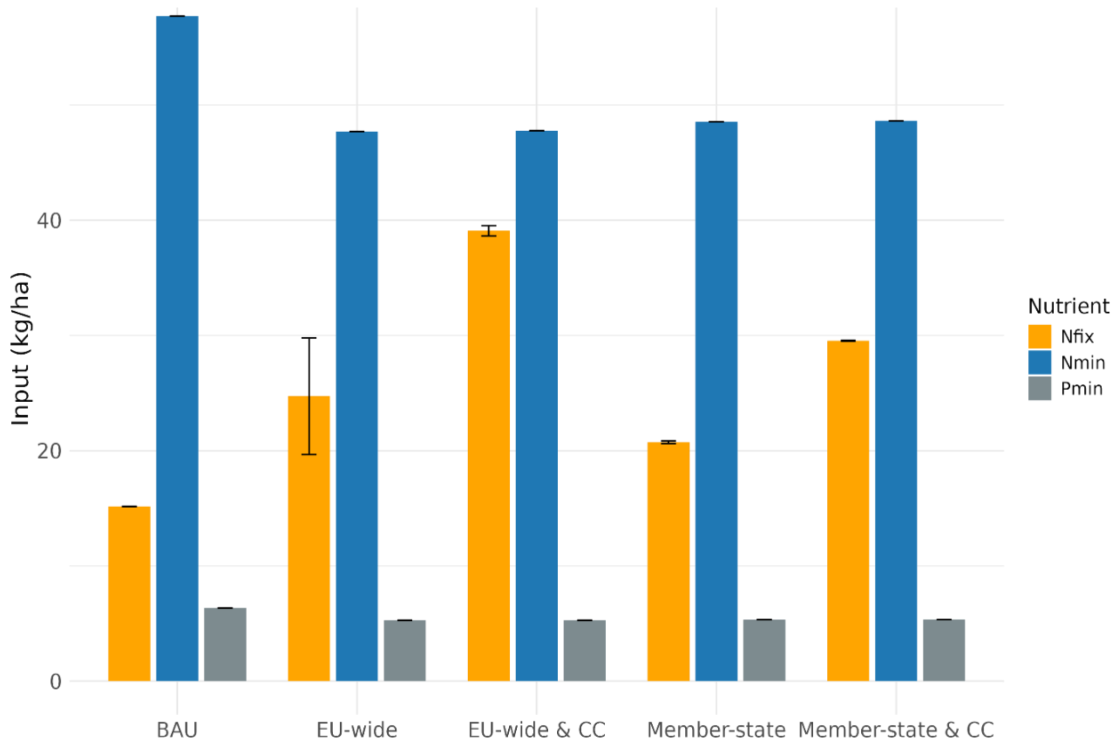

Figure S5. Biological N fixation (Nfix), mineral N (Nmin), and mineral P (Pmin) inputs across scenarios.

Table S8. Global species loss of BAU compared to 25% OA implemented EU-wide or per member state, with or without additional cover crops (CC). The table details impacts for each nutrient loss process and total impacts, presenting both average and marginal impacts as outlined in the methods section. The "±" refers to the range of the impact of the low and high yield gap scenario.

	**CF**	**Nerod** [PDF·year]	**Nleach** [PDF·year]	**Perod** [PDF·year]	**Pleach** [PDF·year]	**Total** [PDF·year]
BAU	average	3.24E-05	3.86E-04	5.26E-05	1.31E-06	4.72E-04
25% OA: EU-wide	average	3.16E-05 ± 1.27E-07	3.93E-04 ± 6.88E-06	4.53E-05 ± 6.54E-06	1.11E-06 ± 1.91E-07	4.71E-04 ± 1.53E-08
	Δmarginal	3.68E-05 ± 7.19E-08	4.29E-04 ± 7.69E-06	7.16E-05 ± 5.09E-07	1.79E-06 ± 2.24E-08	5.39E-04 ± 7.09E-06
25% OA: EU-wide & CC	average	3.12E-05 ± 1.66E-07	4.53E-04 ± 1.49E-05	3.81E-05 ± 8.08E-08	9.44E-07 ± 3.00E-09	5.24E-04 ± 1.46E-05
	Δ marginal	3.64E-05 ± 1.53E-07	4.93E-04 ± 1.29E-05	6.97E-05 ± 1.86E-07	1.83E-06 ± 3.68E-09	6.01E-04 ± 1.25E-05
25% OA: member-state	average	3.22E-05 ± 2.13E-08	3.76E-04 ± 7.12E-06	4.53E-05 ± 6.53E-06	1.11E-06 ± 1.92E-07	4.55E-04 ± 3.81E-07
	Δ marginal	3.74E-05 ± 2.73E-08	4.13E-04 ± 8.92E-06	7.17E-05 ± 4.91E-07	1.79E-06 ± 2.40E-08	5.23E-04 ± 8.44E-06
25% OA: member-state & CC	average	3.21E-05 ± 2.97E-08	4.38E-04 ± 1.40E-05	3.82E-05 ± 4.46E-08	9.45E-07 ± 2.46E-09	5.09E-04 ± 1.39E-05
	Δ marginal	3.73E-05 ± 2.57E-08	4.83E-04 ± 1.44E-05	7.00E-05 ± 1.41E-07	1.83E-06 ± 2.19E-09	5.93E-04 ± 1.42E-05
